# Bridging the performance–efficiency gap: a resource-efficient hybrid DL-ML framework for jackfruit disease detection

**DOI:** 10.3389/frai.2026.1884567

**Published:** 2026-10-06

**Authors:** Rittik Chandra Das Turjy, Fahmid Al Farid, Md. Hasan Imam Bijoy, Sarbajit Paul Bappy, Amir Sohel, Jia Uddin, Hezerul Bin Abdul Karim

**Affiliations:** 1 Department of Computer Science and Engineering, Daffodil International University, Dhaka, Bangladesh; 2 Centre for Image and Vision Computing (CIVC), Centre of Excellence for Artificial Intelligence, Faculty of Artificial Intelligence and Engineering (FAIE), Multimedia University, Cyberjaya, Malaysia; 3 Berlin School of Business & Innovation (BSBI), Berlin, Germany; 4 AI and Big Data Department, Woosong University, Daejeon, Republic of Korea

**Keywords:** agricultural disease detection, custom CNN, ensemble model, feature extraction, machine learning, PCA, SMOTE, jackfruit disease

## Abstract

Complex environment variability and limited computational resources in agricultural settings create challenges in automated detection in tropical regions, where jackfruit diseases pose major threats to harvest productivity and food security. Conventional machine learning models often fail to generalize across diverse image conditions. For sustainable, field-deployable diagnostic systems, it is essential to close the performance–efficiency gap, as traditional deep learning methods require large amounts of training data and high-end hardware. We propose a hybrid deep learning machine learning (DL-ML) framework that has been tailored for accurate and resource-efficient jackfruit disease identification. This framework discusses deep visual representations that are extracted using a lightweight custom Convolutional Neural Network (CNN) combined with a previously trained EfficientNet-B0, capturing both localized and nested image features. Principal Component Analysis (PCA) is utilized for feature optimization to enhance discriminative power and eliminate redundancy, while SMOTE minimizes dataset imbalance by generating synthetic minority samples. These selected features are then classified by employing a variety of machine learning algorithms, including Random Forest (RF), Support Vector Machine (SVM), XGBoost, CatBoost, and k-Nearest Neighbors (KNN). A stacked ensemble model incorporates the strengths of these classifiers, enhancing generalization and robustness under diverse lighting and background conditions. Experiments across multiple dataset configurations show a noticeable improvement in accuracy from 76.80% on raw data to 99.39% on the PCA-augmented dataset, illustrating that this hybrid ensemble consistently outperforms all individual deep and machine learning baselines. Although the approach is currently tailored to jackfruit disease datasets, its applicability to other crops or stress conditions requires greater domain adaptation studies. However, the model's high accuracy highlights its scalability and utility in low-resource agricultural environments. Additionally, it establishes a cost-effective diagnostic paradigm where efficient ML classification and deep feature extraction collaborate, laying the foundation for next-generation smart farming and precision agriculture systems.

## 1 Introduction

One of the most important fruit crops in Bangladesh, Jackfruit (*Artocarpus heterophyllus*), plays a significant role in food security, rural livelihoods, and export earnings in the country. Producing about 2.5 million metric tons every year, it is one of the most widely cultivated tropical fruits in the country, with an area of cultivation of about 150,000 hectares. Jackfruit is essential for the feeding of rural people, providing them the necessary nutrients like carbohydrates, vitamins, and fiber. The adaptability of the fruit, which is used both in its mature and immature forms, makes it a basic ingredient of many native cuisines. Furthermore, it offers economic opportunities to farmers, and in particular to smallholders, through revenue from the domestic market and the growing export markets. In recent years, jackfruit export has increased in Bangladesh as processed products such as canned jackfruit and jackfruit chips have become popular in international markets, thus increasing the economic importance of jackfruit. This aligns with broader trends in agricultural AI, where opportunities such as automated diagnostics are increasingly weighed against persistent deployment challenges; realizing these opportunities in practice requires addressing data scarcity, infrastructure limitations, and model deployability across heterogeneous farming environments (Vocaturo et al., 2023). However, diseases and worsening quality remain an impediment to its production. Automated image-based classification algorithms were developed since the manual inspection of jackfruit leaves and fruits is time-consuming, unreliable, and impracticable for large-scale monitoring (Saha et al., 2024; Haque et al., 2026). Agricultural classification tasks have been solved using traditional machine learning techniques such as Random Forest (RF), Support Vector Machine (SVM), XGBoost, CatBoost, k-Nearest Neighbors (KNN), and ensemble models. These methods are praised for their efficiency and interpretability, but since they are based on manually created descriptors that are not sufficient to capture deep spatial hierarchies in the images, they are not effective when used directly on unprocessed images (A and N, 2023).

Convolutional Neural Networks (CNNs) in particular, are end-to-end deep learning (DL) models that automatically learn discriminative features to achieve superior performance in crop quality grading and fruit disease detection (Vijayan and Chowdhary, 2025; Shafik et al., 2025). Large annotated datasets, significant computational costs, and lengthy training periods are necessary for these models despite their superior precision, which limits their applicability in resource-constrained situations like smallholder farming (Sakka et al., 2025).

As a result, a hybrid alternative has emerged. Shallow ML classifiers like RF, SVM, XGBoost, CatBoost, or KNN are trained on the extracted embeddings under this paradigm, which uses pretrained CNNs or lightweight DL architectures for feature extraction. This approach reduces training costs and computing demands while maintaining DL's representational power. For instance, DL-ML hybrids are more accurate at diagnosing plant diseases than standalone ML or DL methods, who employed lightweight backbones with attention modules to achieve high accuracy under resource constraints, demonstrated that optimized CNN features in conjunction with ML classifiers produce competitive results with reduced complexity in rice crop disease prediction. Rahaman et al. (2025) showed that ML models can map crops with near-DL precision in remote sensing while using far fewer resources. Beyond detection accuracy, recent maize-focused studies have extended deep learning pipelines toward severity grading and crop-loss estimation to provide more actionable outputs for farmers, a customized deep learning framework, MaizeNet, has been proposed to couple K-means based region of interest extraction with disease classification, severity prediction, and crop-loss estimation, achieving 98.50% accuracy on a real world, pathologist labeled maize dataset (Kundu et al., 2022).

Furthermore, recent reviews stress the importance of transfer learning, model compression, and hybrid architectures for realistic agricultural AI. According to Jiang et al. (2025), a scalable route for smart agriculture is provided by effective feature extraction in conjunction with shallow classifiers. An involution-infused DenseNet with two-step compression was proposed by Ahmed et al. (2025), exhibiting resource-efficient plant disease classification without sacrificing accuracy. MobilePlantViT, a mobile-friendly hybrid vision transformer that can classify plant diseases generally on devices with minimal resources, was presented by Tonmoy et al. (2025).

In order to identify jackfruit disease and quality in Bangladesh, our study uses a hybrid approach. This study starts with raw image data for baseline machine learning. Next, this research uses a pretrained EfficientNet-B0 backbone and a custom CNN to extract features. In this study, we use the Synthetic Minority Oversampling Technique (SMOTE) and Principal Component Analysis (PCA) to handle excessive dimensionality and class imbalance. When paired with feature extraction and shallow ML classifiers, these methods have been demonstrated to dramatically increase classification accuracy in a variety of fields, including agriculture (Mulla et al., 2021; Thombre et al., 2023). This research also evaluates the effect of data augmentation. We evaluate RF, SVM, XGBoost, CatBoost, and KNN for classification under different pipeline configurations and suggest an ensemble model that consistently performs better than the individual classifiers. Our goal is to show that hybrid DL-ML pipelines may produce comparable accuracy at a significantly lower computational cost when paired with PCA, SMOTE, and augmentation. These pipelines are feasible to implement in agricultural environments with limited resources.

While conducting this study, several questions arose:

- **RQ1 -** For the raw dataset of jackfruit, how do traditional ML models perform without feature extraction?
- **RQ2 -** Does feature extraction improve classifier accuracy compared to direct ML models?
- **RQ3 -** How do PCA and SMOTE help with problems like class imbalance and high dimensionality?
- **RQ4 -** What effect does data augmentation have on robustness and classification accuracy in different field conditions?
- **RQ5 -** In environments with limited resources, do hybrid DL-ML pipelines offer a better cost-accuracy tradeoff compared to independent ML or DL methods?

To address these questions, this research sets the following objectives:

- **RO1 -** To evaluate the efficacy of conventional ML classifiers on raw jackfruit images.
- **RO2 -** To assess how well CNN-based feature extraction methods improve classification.
- **RO3 -** To evaluate the roles of PCA and SMOTE in addressing imbalance and enhancing efficiency.
- **RO4 -** To investigate the impact of data augmentation in terms of robustness and trustworthiness.
- **RO5 -** To evaluate whether ensemble classifiers perform better compared to single ML models.

The main contributions of this study are summarized below:

- This study constructed a jackfruit image dataset containing six classes. The images were collected under diverse field conditions in Bangladesh. Data augmentation and preprocessing were applied to improve class balance and robustness.
- This research compared three approaches: direct ML on raw images, custom CNN feature extraction with ML, and hybrid DL feature extraction with ML, across both augmented and non-augmented datasets, combined with PCA and SMOTE.
- This study demonstrated that deep feature extraction (custom CNN, EfficientNet-B0) combined with PCA and SMOTE significantly improves the cost-accuracy tradeoff. These hybrid pipelines outperform both direct ML and full DL under constrained resources.
- This study conducted ablation studies to quantify the effects of (a) augmentation, (b) feature extraction methods, (c) dimensionality reduction (PCA), (d) class rebalancing (SMOTE), and (e) ensemble calibration and blending, on model performance metrics (accuracy, precision, recall, F1-score).
- This research highlights the utility of hybrid DL-ML pipelines as resource-efficient solutions for image classification, especially in settings like Bangladesh.

## 2 Related work

There are two major approaches to image-based crop health assessment: deep learning (DL) representations used either end-to-end or as features for shallow ML models, and handcrafted descriptors with classical machine learning (ML) classifiers. Though they require particular crop engineering and deteriorate under changes in illumination or position, handcrafted pipelines are appealing when compute budgets are limited and provide interpretability. In data-scarce agriculture, hybrid “DL feature to ML classifier” designs frequently offer high accuracy at a cheaper training cost. DL models also translate well across various circumstances.

### 2.1 Handcrafted feature baselines and jackfruit

Early studies on jackfruit identification demonstrated how crucial it is to use particular color and texture characteristics for precise identification. For instance, Habib et al. (2020) presented one of the earliest automated methods for jackfruit disease identification by using a Random Forest classifier to evaluate handmade statistical and GLCM characteristics, and k-means clustering to identify diseased spots. The remarkable accuracy of around 90% was attained with this method. Building on these further studies, such as those conducted by Shafik et al. Oraño et al. (2020) and Simangunsong et al. (2025), concentrated on creating affordable solutions appropriate for mobile agronomic support systems. These experiments classified five categories of fruit damage based on field images using trained shallow neural networks and Haralick statistics generated from HSL co-occurrence matrices, achieving an accuracy of 93.42%. These techniques showed potential for effective jackfruit disease identification in real-world, field-based applications, despite mostly depending on manually designed feature extraction and lacking explicit strategies for managing distributional shifts.

### 2.2 Combining ML classifiers with hybrid DL feature extraction

Related agricultural studies have shown that embeddings from pretrained Convolutional Neural Networks (CNNs) with basic machine learning (ML) classifiers can achieve or outperform the performance of end-to-end deep learning models while significantly reducing training time. In order to identify rice leaf disease, for example, Sethy et al. (2020) discovered that CNN features taken from ResNet50, followed by support vector machine (SVM) classification, either matched or exceeded the performance of fine-tuned CNN heads. This suggests that the quality of the representations, rather than the intricacy of the deep classifier, frequently determines accuracy, particularly in situations with limited labeled data. Similarly, Kishore et al. (2022) avoided the requirement for laborious end-to-end retraining by applying SqueezeNet embeddings and multi-class SVM classification to achieve an accuracy of 89.40% on a significant, multi-class maize seed dataset. A similar hybrid approach was also validated in the tomato quality grading domain by Mputu et al. (2024), utilizing Random Forest (RF), SVM, and K-Nearest Neighbors (KNN) classifiers in conjunction with transfer-learned embeddings from InceptionV3 to achieve an impressive 97.5% accuracy without the need for costly fine-tuning. Based on these results, a practical and economical method for agricultural applications is suggested, which includes a compact backbone, feature extraction from the penultimate layer, optional feature compression, and training a linear or kernel-based machine learning classifier.

### 2.3 Compression, augmentation, and feature optimization

In agricultural machine learning, two major technology families are constantly improving hybrid pipelines. In the first, feature optimization and compression are utilized to stabilize shallow model training and reduce redundancy in high-dimensional embeddings. In order to drastically reduce the feature set from SqueezeNet on maize disease detection with the least amount of accuracy loss, Kishore et al. (2022) implemented meta-heuristic feature selection techniques like Bat Algorithm (BA), Whale Optimization Algorithm (WOA), and Grey Wolf Optimizer (GWO). According to their findings, dimensionality reduction techniques (such as Principal Component Analysis, or PCA) can be deployed as an effective and cost-effective preprocessing step prior to classification via Random Forest (RF) or Support Vector Machine (SVM) models when working with high-dimensional embeddings. Similarly, dataset-balancing techniques evaluated alongside supervised learning algorithms for rice and corn yield prediction confirm that balancing strategies meaningfully improve predictive reliability on imbalanced agricultural datasets, a pattern consistent with the recall and F1-score gains observed after applying SMOTE in this study (Di Mauro et al., 2024).

Data augmentation is the second significant enhancement, and it is especially important for small agricultural datasets. The empirical distribution gets better, and the margins for machine learning classifiers are increased when data is augmented via standard geometric and photometric modifications (Lee et al., 2025). This technique is particularly effective in situations where labeled data, such as images of jackfruit disease, is expensive or challenging to acquire due to seasonal restrictions. Augmentation can significantly enhance the performance of machine learning models trained on deep learning features by increasing the amount of dataset that is available.

### 2.4 In search of jackfruit-specific hybrid evidence

The literature of jackfruit classification has progressed through multiple distinct stages. The potential of Convolutional Neural Networks (CNNs) for jackfruit image identification, including the classification of fruit varieties and vegetation, was demonstrated by preliminary research (Sumari et al., 2022; Ginting et al., 2024; Pratondo et al., 2023). Subsequently, research emphasized disease detection, advancing from basic deep learning (DL) models (Tagare et al., 2025; Gomathi et al., 2023) to more intricate, end-to-end architectures. The advanced models, such as the SE-ResNet introduced by Gunasekaran and Thambusamy et al. Gunasekaran and Thambusamy (2025), have attained classification accuracies of 99.25 and 99.50%, respectively.

Although these cutting-edge end-to-end models (Mentari et al., 2025; Gunasekaran and Thambusamy, 2025; Bacalla et al., 2023) show excellent accuracy, they are very computationally expensive and complex, thus pointing out an interesting gap in the jackfruit-specific literature. The domain has shifted from dependence on traditional machine learning (ML) methods (Habib et al., 2020; Oraño et al., 2020) to the deployment of complicated, end-to-end deep learning (DL) models, mainly circumventing the resource-efficient hybrid strategy of “Deep learning feature extraction + shallow machine learning classifier.”

Research in other areas of agriculture suggests that deep learning feature extraction and machine learning classification are competitive or potentially superior and save significantly on training costs, despite the absence of direct comparison to jackfruit. This hybrid technique has shown effectiveness as a cutting-edge technique in the classification of diseases of rice leaves (Sethy et al., 2020) and quality grading of tomatoes (Mputu et al., 2024). In addition, research on maize seed classification (Kishore et al., 2022) and general leaf disease identification (Anari, 2022) has proven this hybrid pipeline to be a robust, scalable, and resource-efficient alternative to comprehensive deep learning methodologies. Consequently, this study aims to cover this gap by conducting the earliest full evaluation of this hybrid deep learning and machine learning pipeline for jackfruit.

### 2.5 Synopsis and research gap

There is a cohesive narrative present in the literature. Although the first attempts at classifying jackfruit used feature classifiers, which initially provided a benchmark for classifying jackfruit (Habib et al., 2020; Oraño et al., 2020), these were quickly surpassed by the development of deep learning (DL) models, which are considerably easier to implement for straightforward end-to-end classification (Gomathi et al., 2023). The contemporary modern technology in jackfruit disease detection has changed into two main methodologies.

#### 2.5.1 Advanced, comprehensive deep learning models

These end-to-end models, such as SE-ResNet (Gunasekaran and Thambusamy, 2025), are tailored for jackfruit disease diagnosis and have attained near-perfect accuracy of 99.25% (Gunasekaran and Thambusamy, 2025). Nevertheless, they are resource-intensive and intricate, necessitating considerable training resources and specialized hardware (Gunasekaran and Thambusamy, 2025; Mentari et al., 2025).

#### 2.5.2 Hybrid deep learning and machine learning pipelines

The “Deep learning feature extraction + shallow machine learning classifier” methodology has demonstrated a high degree of accuracy and resource efficiency across multiple agricultural sectors, including rice (Sethy et al., 2020), corn (Kishore et al., 2022), tomato (Mputu et al., 2024), and general leaf disease classification (Anari, 2022).

This highlights a significant deficiency in the scientific literature. Although the resource-efficient deep learning feature and machine learning pipeline have been shown to be effective in other crops, they remain predominantly unexplored and unmeasured in relation to jackfruit disease detection.

For further clarification, Table 1 is provided below.

**Table 1:** Summary of jackfruit-related studies.

Author(s)	Images	Classes	Best model	Hybrid DL+ML	Ensemble	Accuracy	Remarks
Sumari et al. (2022)	1,000	2	VGG16 (SDG)	✗	✗	96.3%	Jackfruit vs. cempedak fruit species classification.
Oraño et al. (2020)	380	5	BPNN (hand)	✗	✗	93.4%	Handcrafted texture features with pure ML classifiers.
Pratondo et al. (2023)	204	2	VGG16	✗	✗	97.6%	Leaf type classification (jackfruit vs. cempedak).
Gomathi et al. (2023)	600	3	Custom CNN	✗	✗	95.0%	CNN-based tree disease detection (leaf, fruit, trunk).
Tagare et al. (2025)	N/A	4	CNN vs. RF	✗	✓	94.0%	Study showing DL outperforms ML for leaf disease.
Gunasekaran and Thambusamy (2025)	13,245	3	Imp. ResNet	✗	✗	99.3%	Leaf disease detection using attention mechanism.
Bacalla et al. (2023)	N/A	2	ULBP+SVM	✗	✗	93.3%	Maturity classification using binary patterns and SVM.
Habib et al. (2020)	480	5	RF	✗	✓	89.6%	Feature-engineering with ML classifiers for disease.

## 3 Methodology

This study collected and analyzed jackfruit leaf and fruit images from multiple regions and conditions to capture the natural variability of disease and health states. Because reliable field deployment requires well-balanced, representative training data, preprocessing and augmentation were applied systematically at every stage, from image collection through data distribution, as summarized in Figure 1. This design directly supports the study's practical goal: a diagnostic pipeline accurate enough for reliable use, yet lightweight enough to run on the modest imaging equipment and limited connectivity typical of smallholder farms.

**Figure 1:**
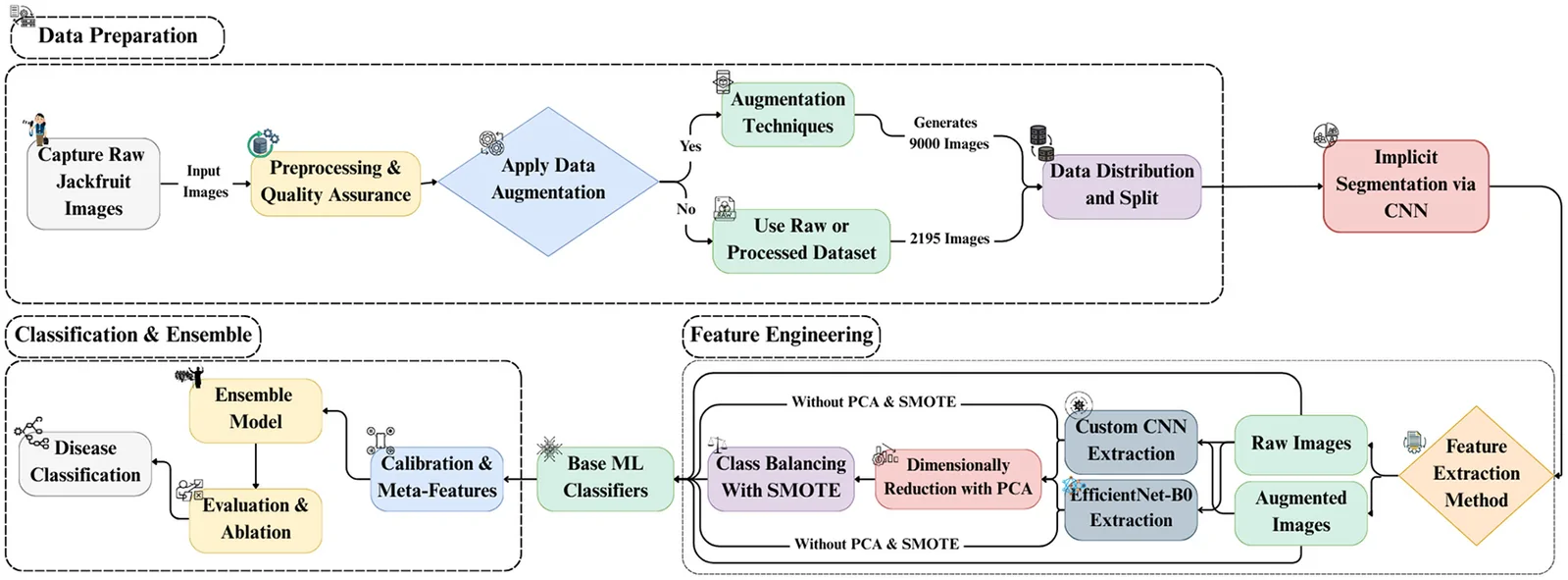
End-to-end workflow of the proposed hybrid DL-ML pipeline, from raw image acquisition and augmentation through feature extraction (custom CNN/EfficientNet-B0), PCA-based dimensionality reduction, SMOTE-based class balancing, base classifier training, and calibrated ensemble fusion.

### 3.1 Data collection and description

A number of jackfruit fields in the surrounding areas were photographed in high quality in order to provide a comprehensive dataset. The purpose of this data gathering technique was to demonstrate the potential diversity of jackfruit health and disease circumstances in real-world farming settings. A OnePlus 7 Pro (OnePlus Technology Co., Ltd., Shenzhen, China) smartphone's 48 megapixel camera with 2160p resolution was used to take the images and an iPhone 12 (Apple Inc., Cupertino, CA, USA) provided extra high-quality photographs. To take into consideration changes in the environment, lighting, and prevalence of disease, this study sampled different areas of Barishal and Khulna at different times of the year.

The collections include 2,195 photos primarily featuring jackfruit leaves and fruits. The images are categorized into six groups: fruit damage (322 images), leaf blight (275 images), rhizopus rot (255 images), healthy leaf (557 images), healthy jackfruit (497 images) and leaf spot (289 images). The images were taken from several jackfruit trees in Barishal and Khulna, Bangladesh for the sake of fairness. The collection documents variations in leaf and fruit conditions which reflect the inherent variability in jackfruit health and disease states. The high-resolution images provide comprehensive insights into the visual characteristics associated with different classes which helps deep learning models be trained and evaluated more effectively. A description of each of the classes listed in Table 2, is provided in Figure 2, along with images of the various jackfruit leaf and fruit diseases (a–f) classes.

**Table 2:** Jackfruit image classes and descriptions.

Class	Description
Fruit damage	Depicts jackfruit that has outward damage from diseases, pests or environmental conditions.
Rhizopus rot	Shows blackened decomposing areas caused by Rhizopus mold.
Healthy jackfruit	Illustrates a jackfruit that is fully healthy with no visible defects or disease signs.
Leaf blight	Discolored necrotic patches caused by bacterial or fungal diseases.
Leaf spot	Leaves with round or irregular spots resulting from infections.
Healthy leaf	Displays fresh, undamaged leaves indicating a healthy plant.

**Figure 2:**
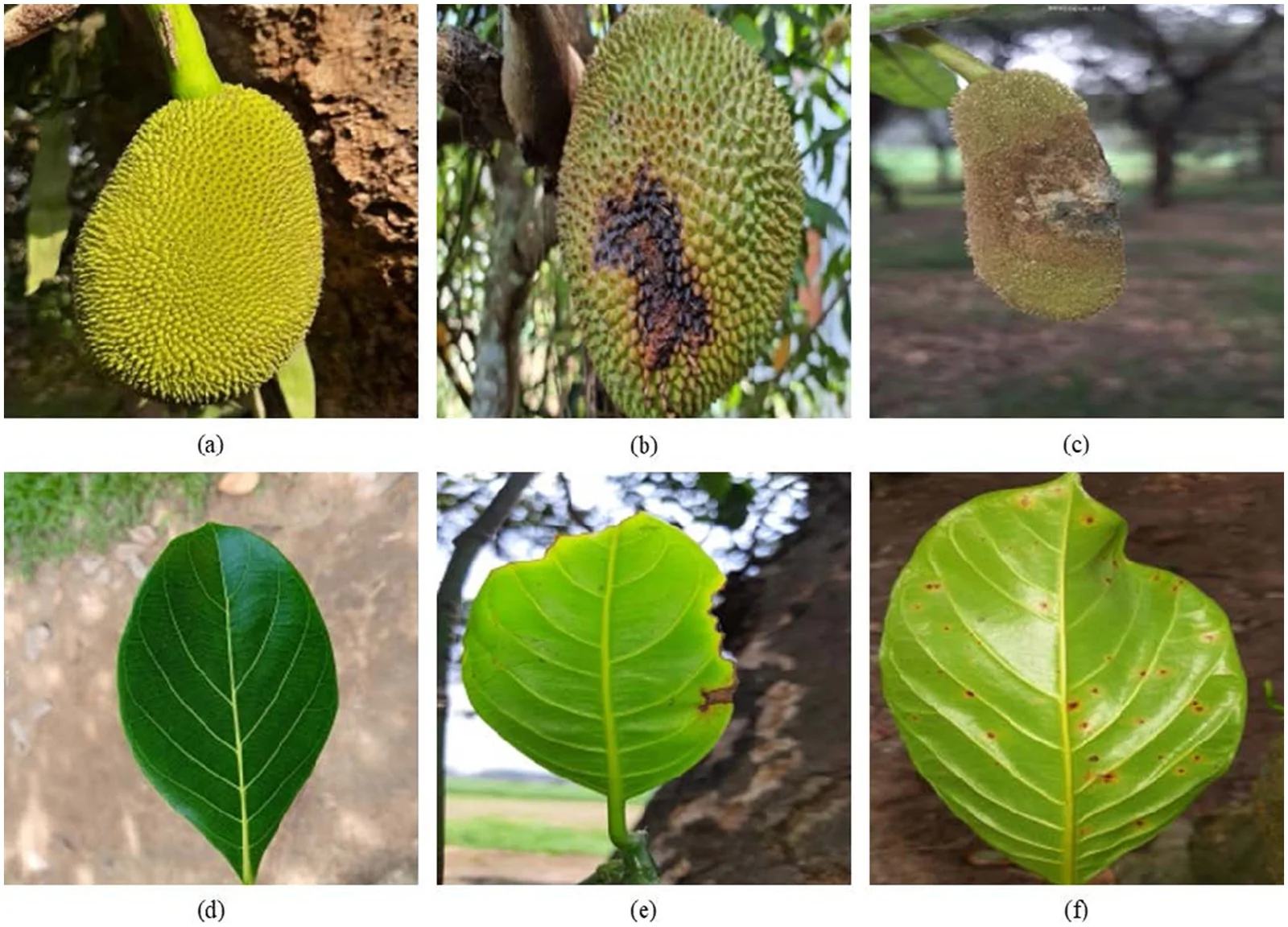
Examples of jackfruit dataset images: **(a)** healthy jackfruit, **(b)** fruit damage, **(c)** rhizopus rot, **(d)** healthy leaf, **(e)** leaf blight, **(f)** leaf spot.

### 3.2 Feature engineering

Feature engineering is the process of converting processed image data into a structure that machine learning models can use more effectively. A bespoke CNN was used in this work to extract features which were then followed by dimensionality reduction and class balancing methods like PCA and SMOTE.

#### 3.2.1 Feature extraction using custom CNN

The custom CNN is a lightweight neural network designed specifically for feature extraction from raw jackfruit images. Each of its three convolutional blocks is succeeded by layers for batch normalization and max pooling. A 256-dimensional feature vector that condenses the most important elements of the image is created by passing the last convolutional layer output through a Global Average Pooling (GAP) layer.

CNN architecture details: the architecture of the custom CNN is built as a deep feature extractor and involves multiple convolutional blocks whose architecture is designed to remove the redundant spatial information from raw data into more discriminative data. The model's embedded output space transitions from low level edges to complex semantic patterns, creating a powerful 256 dimensional embedding specially optimized for the classification of jackfruit.

The structural specifications and functional roles of each layer within this pipeline are detailed in Table 3.

**Table 3:** Architectural specifications of the custom CNN feature extractor.

Component	Configuration	Functional description
Convolutional blocks	32, 64, 128 filters	Sequential feature extraction via increasing filter depth.
Pooling layers	Max-pooling	Dimensionality reduction while preserving dominant spatial features.
Aggregation	Global average pooling	Consolidation of spatial data into a localized feature vector.
Output layer	Dense (256-D)	Generation of high-dimensional embeddings for visual pattern discrimination.

#### 3.2.2 Dimensionality reduction with PCA

Principal Component Analysis (PCA) was used to reduce the 256-dimensional feature vectors in order to further refine the feature set and reduce the chance of overfitting. By reducing less significant elements PCA finds the principal components which most effectively capture the dataset's variation.

PCA statistics: the dimensionality reduction process aims at reducing the feature space to capture the most relevant variance while allowing to reduce noise and redundancy. The high dimensional embeddings are then reduced to a more compact representation, based on Principal Component Analysis (PCA), which greatly improves both the computational efficiency and the classification accuracy. The following description summarizes the impact of the transformation, followed by the technical specifications in Table 4.

**Table 4:** Impact of PCA-based dimensionality reduction on feature space and model performance.

Metric	Original state	Post-PCA (64 components)
Feature dimensionality	256 dimensions	64 dimensions
Information retention	100% variance	95% explained variance
Overall accuracy	Baseline	+3.5% improvement
Minority class recall	Baseline	+5.0% improvement

#### 3.2.3 SMOTE for class balancing

In order to address the class imbalance, we created synthetic samples for the minority classes using the Synthetic Minority Oversampling Technique or SMOTE. This strategy improves the model's capacity to classify diseases that are underrepresented in the training set.

SMOTE impact: class distribution prior to SMOTE showed that healthier classes, such as healthy jackfruit, had significantly more samples than those affected by rhizopus rot and leaf spot; however, following the application of SMOTE, class distributions were balanced through the generation of synthetic examples for underrepresented categories, which improved model generalization. Consequently, recall for minority classes improved by 13%, while the F1-score for these classes increased by 10% after implementing the SMOTE technique.

### 3.3 Data augmentation

One essential technique that improves model resilience and generalization is image augmentation, which is especially helpful when working with small datasets. The model becomes more equipped to learn features and the chance of overfitting is decreased by adding a variety of variants to the data. The deep learning model operates dependably on unseen data thanks to the simulation of real-world changes in perspective, lighting, and orientation (Lee et al., 2025).

Table 5 below lists the augmentation techniques and their parameters, demonstrating the effectiveness of data augmentation in the suggested systems. This study significantly enhances the dataset's quality and diversity by utilizing these data enrichment strategies. This guarantees that our extracted feature is not only well-prepared but also incredibly flexible in order to identify jackfruit diseases in a variety of settings.

**Table 5:** Data augmentation parameters.

Parameter	Values
Rotation	45°, 60°
Horizontal and vertical flipping	Enabled
Shear factors	5, –5
Skew factors	0.1, –0.1
Brightness	1.2

### 3.4 Image quality evaluation metrics

To assess whether data augmentation and preprocessing caused any negative artifacts that would bias feature representations learned downstream, image fidelity was quantitatively measured using six standard measures: Peak Signal-to-Noise Ratio (PSNR) (Fardo et al., 2016), Structural Similarity Index Measure (SSIM) (Peng et al., 2020), Multi-Scale Structural Similarity (MS-SSIM) (Wang et al., 2003), Mean Squared Error (MSE) (Wang and Bovik, 2009), Root Mean Squared Error (RMSE) (Hodson, 2022), and Visual Information Fidelity (VIF) (Sheikh and Bovik, 2006). Together, they offer a comprehensive evaluation based on several aspects: pixel error, multi-scale structural coherence, and perceptual information retention.

A representative validation subset of twelve randomly sampled images per class (Table 6) was used to evaluate the quality after augmentation. The pipeline showed high perceptual and structural fidelity, with the PSNR values being grater then 24dB, SSIM scores in the range of 0.860–0.950 and MS-SSIM scores in the range of 0.976–0.982. Likewise, the range of MSE (0.001–0.018) and RMSE (0.034–0.041) values and the VIF scores (0.497–0.755) indicated good visual information preservation in most categories. For extreme cases of localized degradation (mainly in Healthy Leaf class) samples were systematically reprocessed or pruned to ensure the integrity of the dataset and avoid artifact propagation during model training.

**Table 6:** Image quality metrics for selected images.

Image	PSNR	SSIM	MS-SSIM	MSE	RMSE	VIF
1	27.26	0.886	0.977	122.11	11.05	0.497
2	27.20	0.876	0.977	123.82	11.13	0.580
3	25.52	0.913	0.982	182.44	13.51	0.573
4	27.10	0.916	0.980	126.74	11.26	0.627
5	26.96	0.860	0.976	131.02	11.45	0.507
6	21.83	0.950	0.975	426.59	20.65	0.755
7	28.05	0.908	0.983	101.80	10.09	0.612
8	27.50	0.913	0.982	115.67	10.76	0.610
9	26.64	0.891	0.982	140.87	11.87	0.656
10	27.18	0.903	0.981	124.44	11.16	0.605
11	27.28	0.903	0.982	121.76	11.03	0.587
12	28.38	0.940	0.981	94.44	9.72	0.707

### 3.5 Data distribution

The initial 2,195 primary photos for each illness category were subjected to a variety of augmentation approaches following the completion of the augmentation preprocessing step yielding 9,000 images. Three datasets (raw, preprocessed, and augmented) were prepared for thorough model validation. The dataset was separated into training, validation, and testing subsets: 70% training, 10% validation, 20% testing, which is a frequently utilized split ratio for effective and impartial machine learning model training (Xu and Goodacre, 2018). Table 7 displays the comprehensive data distribution among the various categories.

**Table 7:** Data distribution by class.

Class	Primary	Augmented	Train	Validation	Test
Fruit damage	322	1,500	1,050	150	300
Rhizopus rot	255	1,500	1,050	150	300
Healthy jackfruit	497	1,500	1,050	150	300
Leaf blight	275	1,500	1,050	150	300
Leaf spot	289	1,500	1,050	150	300
Healthy leaf	557	1,500	1,050	150	300
**Total**	**2,195**	**9,000**	**6,300**	**900**	**1,800**

Bold values indicate totals.

### 3.6 Data partitioning and anti-leakage protocol

To establish high confidence in the empirical validity of our proposed framework and systematically eliminate the risk of overfitting or data leakage, a rigorous data separation protocol was enforced. The complete dataset was originally organized into clean, class-specific subfolders. Prior to executing any spatial augmentations, minority-class oversampling, or feature extraction procedures, a stratified train/validation/test split was executed directly on the raw files using fixed allocation ratios (70% training, 10% validation, and 20% testing).

Crucially, the out of sample test partition, consisting of 1, 800 untouched field samples was completely isolated from the SMOTE plus Tomek boundary balancing block. This partition serves purely as an independent benchmark. By maintaining a strict barrier between the feature alignment phase and the target evaluation data, we ensure that the high classification accuracies reported are driven by genuine visual indicators rather than artifact replication or shared training statistics.

### 3.7 Feature extraction architecture

The proposed feature extraction method was designed to generate deterministic, high-throughput image embeddings from the dataset. Depending on configuration, the framework can support either a transfer-learning-based EfficientNet-B0 backbone or a compact custom CNN. All experiments were conducted using TensorFlow with GPU acceleration, ensuring numerical stability and reproducibility across runs.

#### 3.7.1 Pipeline overview

The five main steps of the entire pipeline are feature extraction, model initialization, preprocessing, dataset ingestion, and storage. An efficient tf.data input pipeline is used to analyze the images after they have been automatically obtained from class-wise subdirectories under the dataset root. In accordance with the active backbone type, every image undergoes decoding, antialiasing, resizing to 224 × 224 pixels, RGB conversion, and normalization. The preprocess_input normalization [scaling to (−1, 1)] is used for EfficientNet-based extraction, while the custom CNN uses pixel values scaled to [0, 1].

#### 3.7.2 Reproducibility and precision control

To ensure reproducibility, deterministic computation was imposed using TensorFlow experimental determinism mode and fixed seeds (SEED = 42). TensorFlow's mixed_float16 policy was used to enable mixed precision training on GPUs. To avoid precision drift during feature export, activations were saved in float16 while the dense head was kept in float32. Prior to model serialization, every operation in the feature extraction layer was manually cast back to float32.

#### 3.7.3 Feature extraction and export

To process image batches of size 64, GPU-accelerated forward inference was implemented. The feature model output was collected as a 256-dimensional embedding per image for each batch. After being gathered into a Pandas DataFrame, the extracted features were exported in CSV and Parquet formats.

Figure 3 illustrates the architecture of the proposed CNN based feature extraction pipeline.

**Figure 3:**
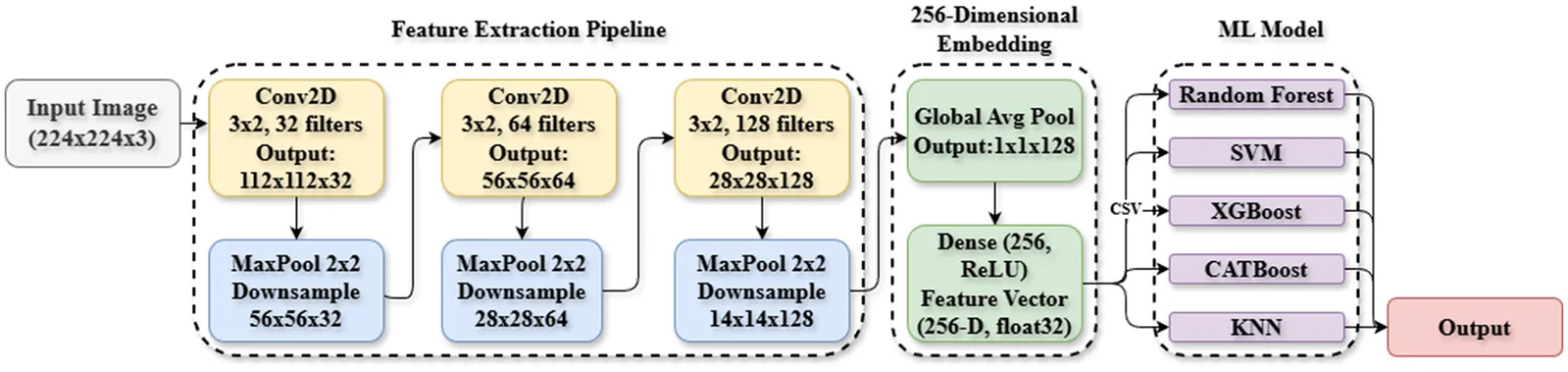
Proposed feature extraction architecture for robust jackfruit detection frameworks.

### 3.8 Description of conventional ML models

This study incorporated five machine learning classifiers: Random Forest (RF), Support Vector Machine (SVM), eXtreme Gradient Boosting (XGBoost), Categorical Boosting (CatBoost), and k-Nearest Neighbors (KNN). Each of these standard algorithms was selected for its proven efficiency in handling high-dimensional feature embeddings. The hyperparameter search grids and the final optimal configurations selected for each base classifier are summarized in Table 8.

**Table 8:** Hyperparameter search grids and selected optimal configurations for each model.

Model	Parameter grid	Optimal settings
RF	*n*_*estimators*: [100, 200, 300] *max*_*depth*: [10, 15, 20] *max*_*features*: [sqrt, log2] *min*_*samples*_*split*: [2, 5] *min*_*samples*_*leaf*: [1, 2]	*n*_*estimators* = 300 *max*_*depth* = 20 *max*_*features* = log2 *min*_*samples*_*split* = 2 *min*_*samples*_*leaf* = 1
SVM	*C*: [0.01–1,000] γ: [1 × 10^−5^–10] Kernel: RBF	*C* = 15.20 γ = 0.00316
XGBoost	*n*_*estimators*: [1,000, 2,000] *learning*_*rate*: [0.03, 0.06] *max*_*depth*: [3–5] *min*_*child*_*weight*: [1, 3] *subsample*: [0.8, 1.0] *colsample*_*bytree*: [0.8, 1.0]	*n*_*estimators* = 2,000 *learning*_*rate* = 0.06 *max*_*depth* = 5 *min*_*child*_*weight* = 1 *subsample* = 0.8 *colsample*_*bytree* = 0.8
CatBoost	*iterations*: [2000] *learning*_*rate*: [0.03, 0.06] *depth*: [4–6] *l*2_*leaf*_*reg*: [1–5] *bagging*_*temperature*: [0.0, 1.0]	*iterations* = 2,000 *learning*_*rate* = 0.06 *depth* = 6 *l*2_*leaf*_*reg* = 1.0 *bagging*_*temperature* = 1.0
KNN	*n*_*neighbors*: [3, 5, 9, 15] *metric*: [euclidean, cosine] *weights*: [distance] *leaf*_*size*: [30]	*n*_*neighbors* = 1 *metric* = cosine *scaler* = StandardScaler *weights* = uniform

### 3.9 Description of ensemble model

The proposed framework implements a multi-stage ensemble learning and calibration pipeline designed to enhance both predictive accuracy and probabilistic reliability. The methodology integrates stratified k-fold ensembling, temperature scaling, and meta model stacking with feature-level calibration, resulting in robust and interpretable performance across validation and test partitions. Figure 4 provides a visual overview of the pipeline. The proposed ensemble framework follows a five-stage pipeline: (i) stratified cross-validation and data partitioning, (ii) base model training, (iii) out-of-fold probability collection, (iv) probability calibration and meta-feature construction, and (v) final ensembling via stacking and weighted blending. Each stage is isolated to prevent information leakage while enabling reliable uncertainty estimation.

**Figure 4:**
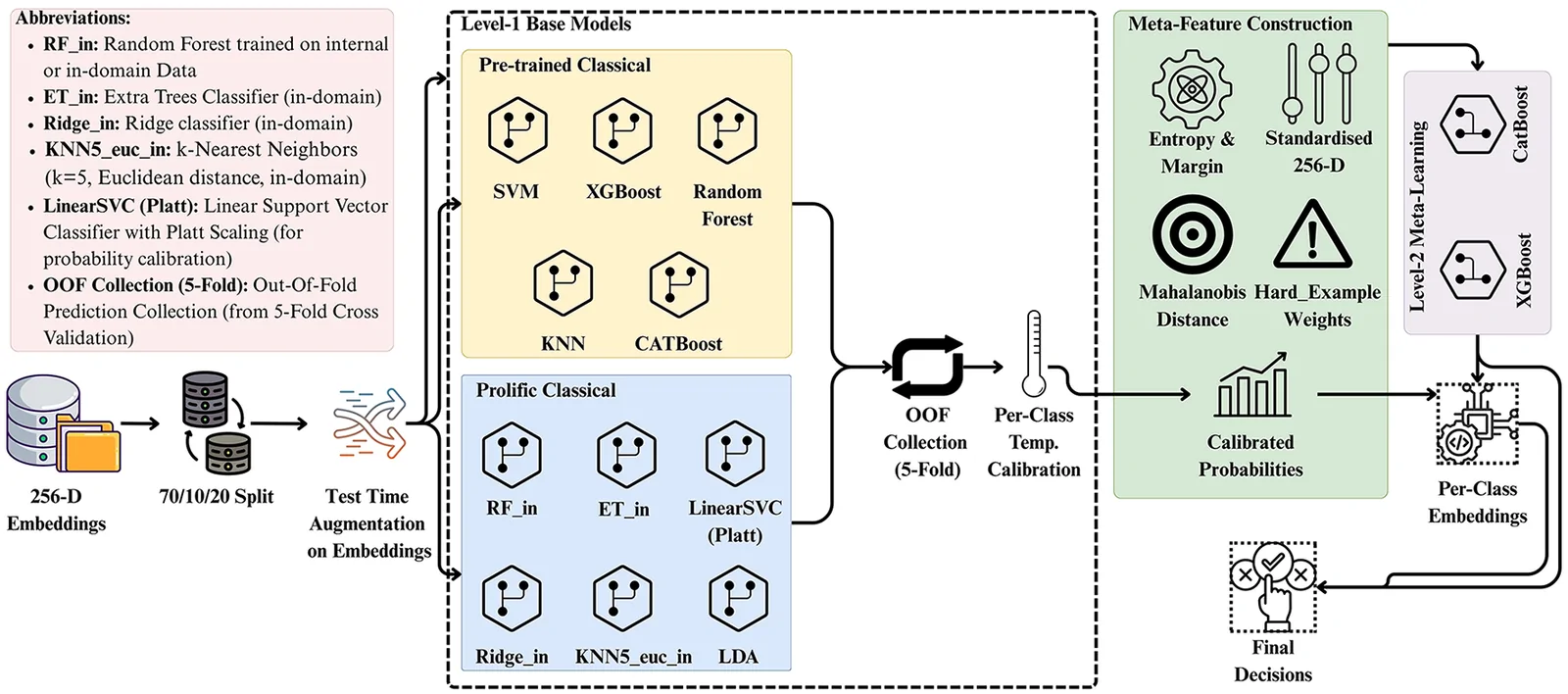
Proposed ensemble model architecture combining multiple classifiers for improved prediction accuracy.

#### 3.9.1 Data partitioning and cross validation

The extracted feature vectors and labels are processed using five-fold stratified cross-validation to preserve class distribution across folds. In each fold, the training subset develops fold-specific base classifiers, while pre-trained classical models remain fixed; the held-out out-of-fold (OOF) subset is reserved exclusively for generating class-probability estimates used in calibration, meta-feature construction, and ensemble fusion. This design supports reliable model evaluation and consistent estimation of prediction uncertainty.

#### 3.9.2 Base model training and out-of-fold probability aggregation

The ensemble framework employs two complementary groups of classical machine learning base models operating on fixed feature embeddings. Pretrained base models such as [Random Forest (RF), Support Vector Machine (SVM), XGBoost (XGB), CatBoost and k-Nearest Neighbors (KNN)] are trained once using the full training dataset with fixed preprocessing pipelines to provide globally stable class probability estimates, whereas the fold-specific base models (the same set of classifiers) are retrained independently within each fold of the cross-validation procedure to generate unbiased out-of-fold (OOF) probability predictions. This fold-wise training captures data variability across splits and supports reliable calibration and meta-learning for downstream ensembling. For each base model, OOF class probability predictions obtained across all folds are aggregated to form a comprehensive probabilistic representation for every training sample. These aggregated OOF probabilities serve as the foundation for probability calibration, meta-feature extraction, and subsequent ensemble learning.

#### 3.9.3 Temperature calibration and meta-feature engineering

Class-wise temperature scaling is applied to the predicted class probabilities to improve probabilistic calibration. Optimal temperature parameters are learned using OOF predictions and subsequently applied consistently to validation and test predictions. This calibration step reduces confidence miscalibration and enhances reliability, which is critical for high-stakes decision-making scenarios. From the calibrated base model probabilities, three groups of meta-features are constructed. The primary group comprises entropy and margin features, which quantify predictive uncertainty and inter-class separability. The second category encompasses on calibrated probability features, which preserve the confidence structure of individual base models. Lastly, prototype distance features, which measure distances between sample probability vectors and class specific prototypes to enhance discrimination.

#### 3.9.4 Meta-model construction and weighted blending

Candidate meta-learners, including XGBoost and CatBoost, are trained on the aggregated meta features. Model selection is guided by validation performance, after which the best-performing meta-model is retrained using all available calibrated OOF predictions. In addition to stacking, a complementary class-wise weighted blending strategy is applied to the OOF predictions. Class-specific weights are optimized to balance the contributions of individual base models, particularly under class imbalance conditions.

## 4 Experimental setup and results analysis

### 4.1 Experimental setup

As this is lengthy experimental research, several environments are needed for parallel execution. Experiments on the augmented dataset were carried out on Kaggle utilizing its high memory compute runtime and TPU v5e-8 accelerator, which offer noticeably prompt training for deep learning workloads. The raw dataset was initially processed on a local Acer Nitro 5 system with an AMD Ryzen 5 5600H CPU, NVIDIA GeForce GTX 1650 GPU, and 24 GB of RAM before being moved to the Kaggle environment for extensive training and assessment.

### 4.2 Result analysis

Performance of models in jackfruit disease classification cannot be measured by numbers alone. It also represents the feasible possibilities for farmers and agronomists. In this research, accuracy and F1 scores are used to measure performance, but they were also concerned with the way such models can respond to real-world variations in leaves and fruits. We are able to analyze detailed metrics and integrate a combination of high-level methods such as ensemble learning, feature extraction, PCA, and SMOTE, to understand which methods are strong, reliable, and significant in the real-world disease detection in jackfruit farming. The next parts confirm how various models and strategies perform through a variety of experimental designs.

#### 4.2.1 Machine learning algorithms in the raw dataset

This research applies Random Forest (RF), Support Vector Machine (SVM), XGBoost (XGB), CatBoost (CB), K-Nearest Neighbors (KNN) and our proposed ensemble model. In this study, we achieved 69.8% accuracy and an F1_macro score of 52.73% in Random Forest (RF). Using Support Vector Machine (SVM), this study obtained an accuracy of about 70.50%, which is a small improvement above Random Forest. Then, we utilized XGBoost (XGB) and CatBoost (CB), which both got 70.20% accuracy and macro scores of 63.00 and 61.10%, respectively. In K-Nearest Neighbors (KNN), we achieved a 69.90% accuracy rate and an F1_macro score of 58.00%. Then, we implemented the proposed ensemble model.

By using this approach, this study achieved the best accuracy of 76.80% with our ensemble model, which is superior to all the pre-trained models. It achieved an F1 macro score of 74.90% and a ROC (Receiver Operating Characteristic) score of 96.10%. For the “Healthy Leaf” class, the highest recall was 86.00%, and for the “Leaf Spot” class, the lower recall was 62.00%. The highest level of precision was 86.00% in the Healthy Leaf class, and the lowest was 67.00% in the Rhizopus Rot class. The “Healthy Leaf” class had an incredible F1 score of 83.00%, while the “Leaf Spot” class had the lowest score of 66.00%. The performance Table 9 is provided below for enhanced comprehension.

**Table 9:** Performance of machine learning algorithms on the raw dataset.

Model	Precision	Recall	F1-score	Accuracy
RF	75.5	74.9	75.0	74.9
SVM	78.3	76.5	77.1	76.6
XGBoost	76.2	75.1	75.5	75.2
CatBoost	75.9	75.0	75.4	75.0
KNN	73.2	72.4	72.7	72.4
**Ensemble model**	**75.5**	**74.9**	**74.98**	**76.8**

Bold values indicate the best-performing model.

The ensemble model's ROC curve and confusion matrix on the raw dataset are shown in Figure 5.

**Figure 5:**
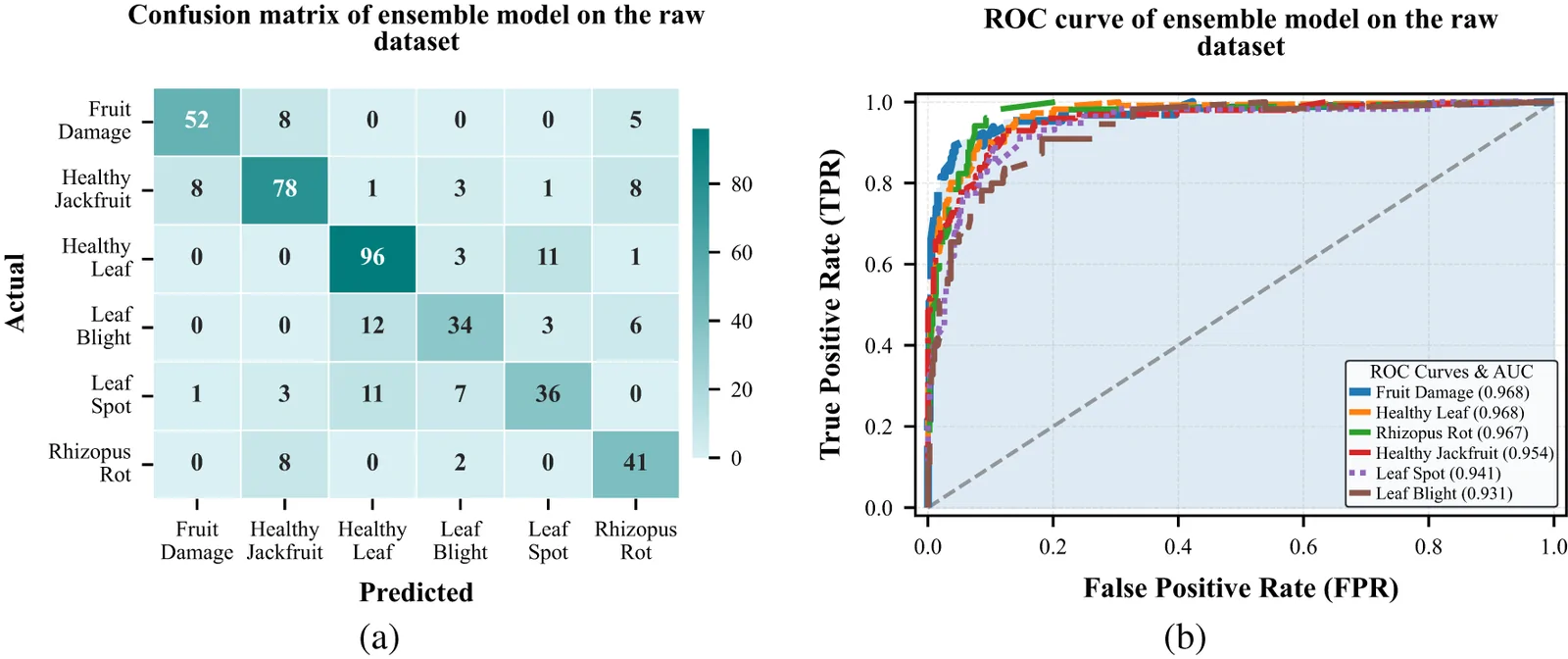
Performance visualization of the ensemble model on the raw dataset: **(a)** confusion matrix and **(b)** ROC curve.

#### 4.2.2 Feature extraction using a custom CNN with machine learning classification

In this study, the extraction of features was performed using two different backbones: an efficient custom convolutional neural network (CNN) and the EfficientNetB0 network. Both models performed the global average pooling followed by a dense layer resulting in a fixed 256-dimensional feature vector. Three batch convolution pooling normalization stages were utilized for the custom CNN and when weights that had been pretrained were available, each one was loaded. In order to guarantee a consistent feature dimension through an extra dense projection layer, EfficientNetB0 was utilized with ImageNet initialization and without the top classification head. For effective GPU inference, the input pipeline processed all of the images in batches after decoding and resizing them to 224 × 224 pixels and properly normalizing them [either by scaling to (0, 1) for the custom CNN or by using EfficientNet preprocessing]. The feature projection layers required float32 to maintain numerical stability, especially when the accuracy was mixed. In order to help with reproducible machine learning experiments afterward, the gathered features were finally merged and stored in both CSV and Parquet formats alongside class labels and file metadata.

In RF, this analysis achieved an accuracy of 72.67%, which in the Fruit Damage class has the highest precision, F1, and recall of 84.60%. With a weighted average of 77.99% and a macro average of 77.61%, in this study, we were able to obtain an accuracy score of 78.13% via SVG. With an accuracy of 71.98%, and the highest precision of 80.88%, recall of 84.62%, and F1 of 82.71%, XGBoost outperforms RF but underperforms SVG. With an accuracy of 68.34%, CatBoost and KNN both perform the lowest out of all the models. Lastly, with an accuracy of 79.75%, the suggested ensemble model performs superior to any other model. Below is the performance comparison of the models, as presented in Table 10, for better understanding.

**Table 10:** Performance of machine learning algorithms with custom CNN feature extraction.

Model	Precision	Recall	F1-score	Accuracy
RF	74.90	72.50	73.20	72.70
SVM	76.80	73.10	74.90	78.10
XGBoost	75.40	72.90	74.20	72.00
CatBoost	74.60	72.80	73.70	68.30
KNN	73.00	71.50	72.20	68.30
**Ensemble model**	**75.00**	**83.10**	**78.09**	**79.80**

Bold values indicate the best-performing model.

Figure 6 present the ROC curve and confusion matrix using custom CNN-extracted features.

**Figure 6:**
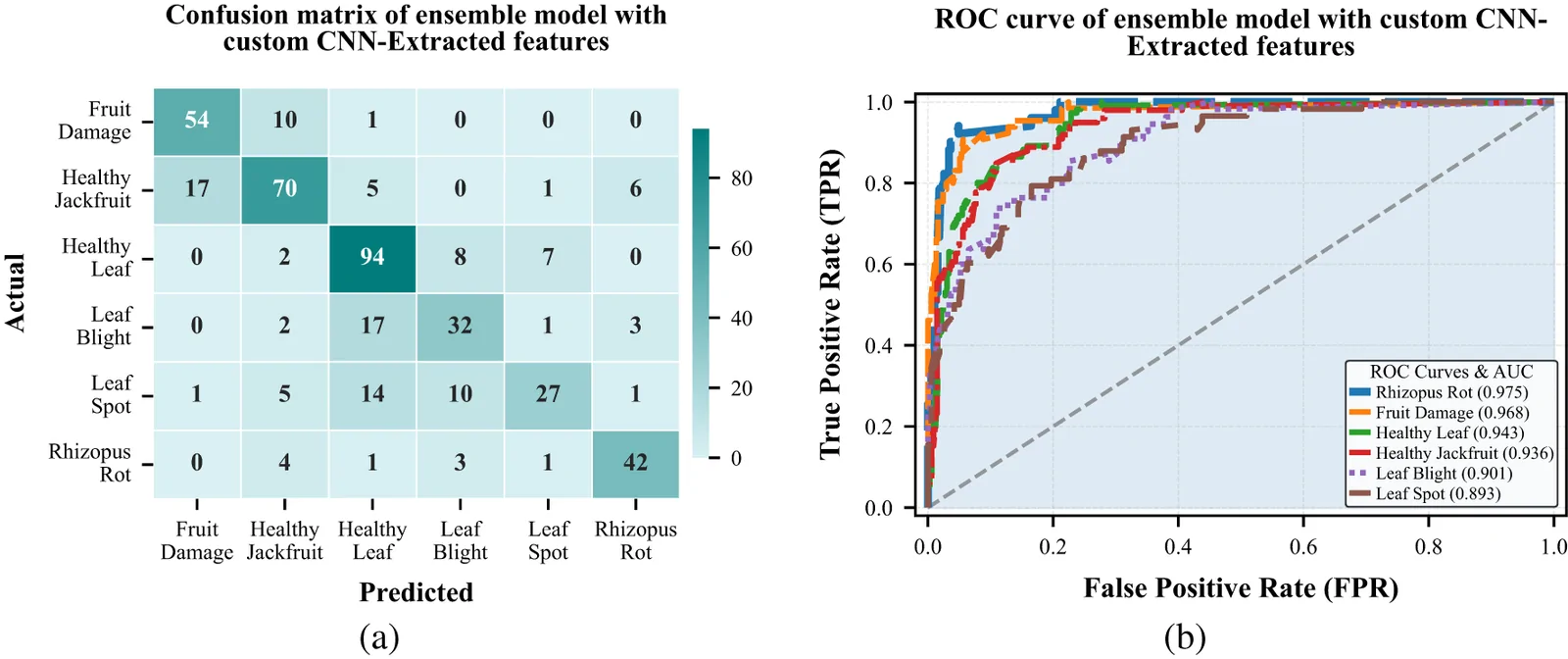
Performance visualization of the ensemble model with custom CNN-extracted features: **(a)** confusion matrix and **(b)** ROC curve.

Even though the custom CNN feature's greatest accuracy increased by 3.84% compared to direct ML, the model is still not performing well for the imbalance in classification. After overcoming this, in this analysis we used PCA and SMOTE to enhance the outcome.

#### 4.2.3 SMOTE-based balancing and PCA reduction of custom CNN features for machine learning models

Following the use of SMOTE and PCA, every model outperforms earlier methods. This study succeeded in attaining 87.10% accuracy in RF, which is the lowest of all the models but still superior to the earlier method. This research obtained an accuracy of 90.30% in SVM, with a precision macro average of 90.69%, the same F1 and recall of 90.40%. This study achieved 87.50% in XGBoost, which is more than RF but less than SVM. This analysis scored 87.10% accuracy in CatBoost, which is the same as RF. Performance in KNN was higher than that of CatBoost and RF by 84.80%. Finally, with an accuracy of 90.74%, the ensemble model outperforms all other models. Its macro average precision, recall, and F1 score are 90.81%, 90.68%, and 90.64%, respectively. And a weighted average of 90.82% for precision, 90.74% for recall, and 90.67% for F1-score. The performance comparison of the models is presented in Table 11 for better comprehension.

**Table 11:** Performance of machine learning algorithms with SMOTE-Based balancing and PCA-reduced custom CNN features.

Model	Precision	Recall	F1-score	Accuracy
RF	88.30	97.20	92.60	87.10
SVM	92.00	95.40	93.70	90.30
XGBoost	89.70	95.40	92.40	87.50
CatBoost	88.40	97.20	92.60	87.10
KNN	86.10	97.20	91.30	84.80
**Ensemble model**	**89.90**	**98.20**	**93.90**	**90.70**

Bold values indicate the best-performing model.

The ROC curve and confusion matrix with SMOTE-balanced and PCA-reduced custom CNN features are given in Figure 7.

**Figure 7:**
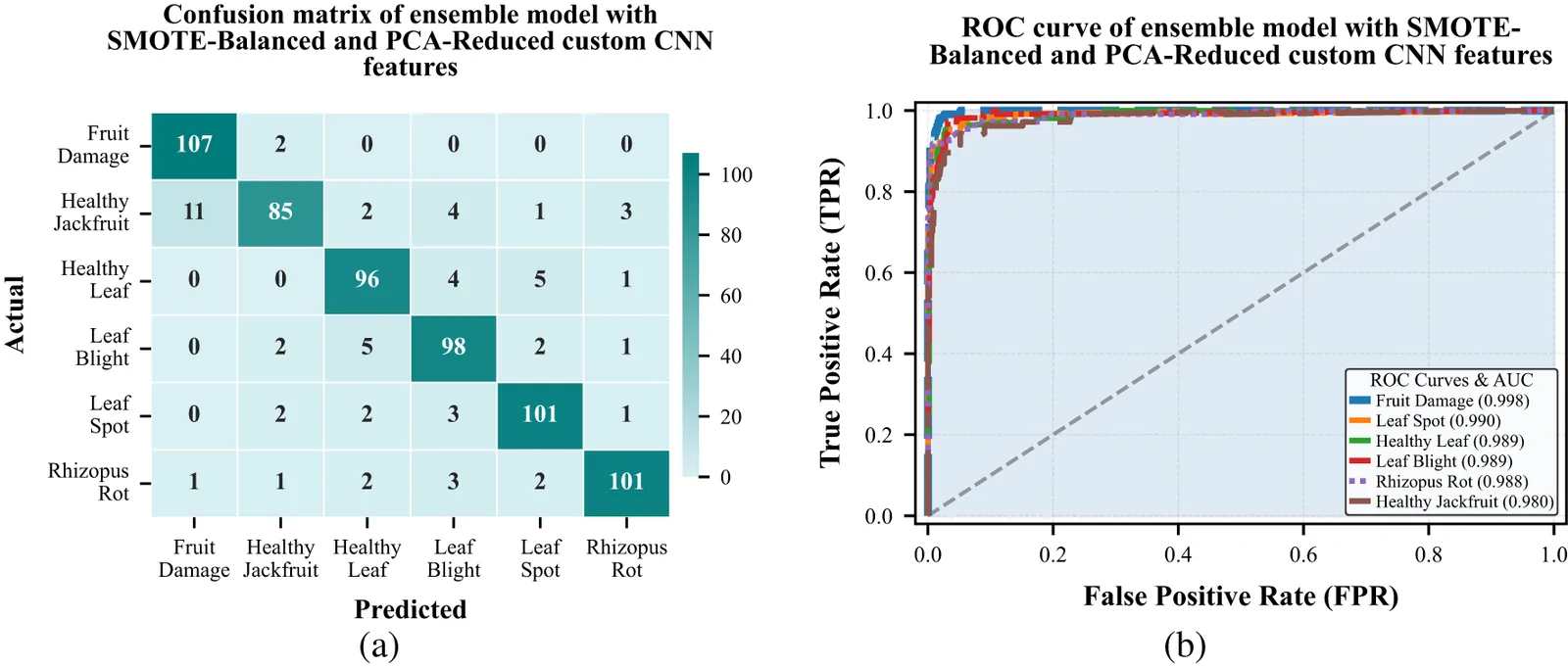
Performance visualization of the ensemble model with SMOTE-balanced and PCA-reduced custom CNN features: **(a)** confusion matrix and **(b)** ROC curve.

Even though the method of applying SMOTE and PCA to customized CNN features has outperformed previous methods, it might be possible to achieve even greater performance by using a pretrained deep learning model to extract features. Better classification performance may result from applying transfer learning from extensive pretrained architectures, which could boost the representational capacity of the extracted features.

#### 4.2.4 Extracted features from EfficientNet-B0 applied to machine learning models

EfficientNet-B0 architecture is able to capture more meaningful features than the customized extracted feature, which is clearly visible in model performance. With macro-averaged precision, recall, and F1 score of 87.97%, 84.37%, and 85.62%, respectively, RF achieved an accuracy of 86.56%. The results are summarized in Table 12. With an accuracy of 89.75%, SVM outperformed. With a macro average of 89.58% precision, 86.45% recall, and 87.60% F1 score, XGBoost achieved an accuracy of 88.61%. CatBoost came in second with macro averages of 88.13% precision, 83.43% recall, 85.21% F1 score, and an accuracy of 85.88%. With a macro-averaged F1 score of 84.80%, the KNN classifier scored an accuracy of 85.88%. Ultimately, with weighted averages of 90.59%, 90.20%, and 90.06.

**Table 12:** Performance of machine learning algorithms with EfficientNet-B0 extracted features.

Model	Precision	Recall	F1-score	Accuracy
RF	87.97	84.37	85.62	86.56
SVM	89.41	88.42	88.66	89.75
XGBoost	89.58	86.45	87.60	88.61
CatBoost	88.13	83.43	85.21	85.88
**Ensemble model**	**91.38**	**88.07**	**89.32**	**90.21**

Bold values indicate the best-performing model.

Figure 8 show the ROC curve and confusion matrix based on EfficientNet-B0 extracted features.

**Figure 8:**
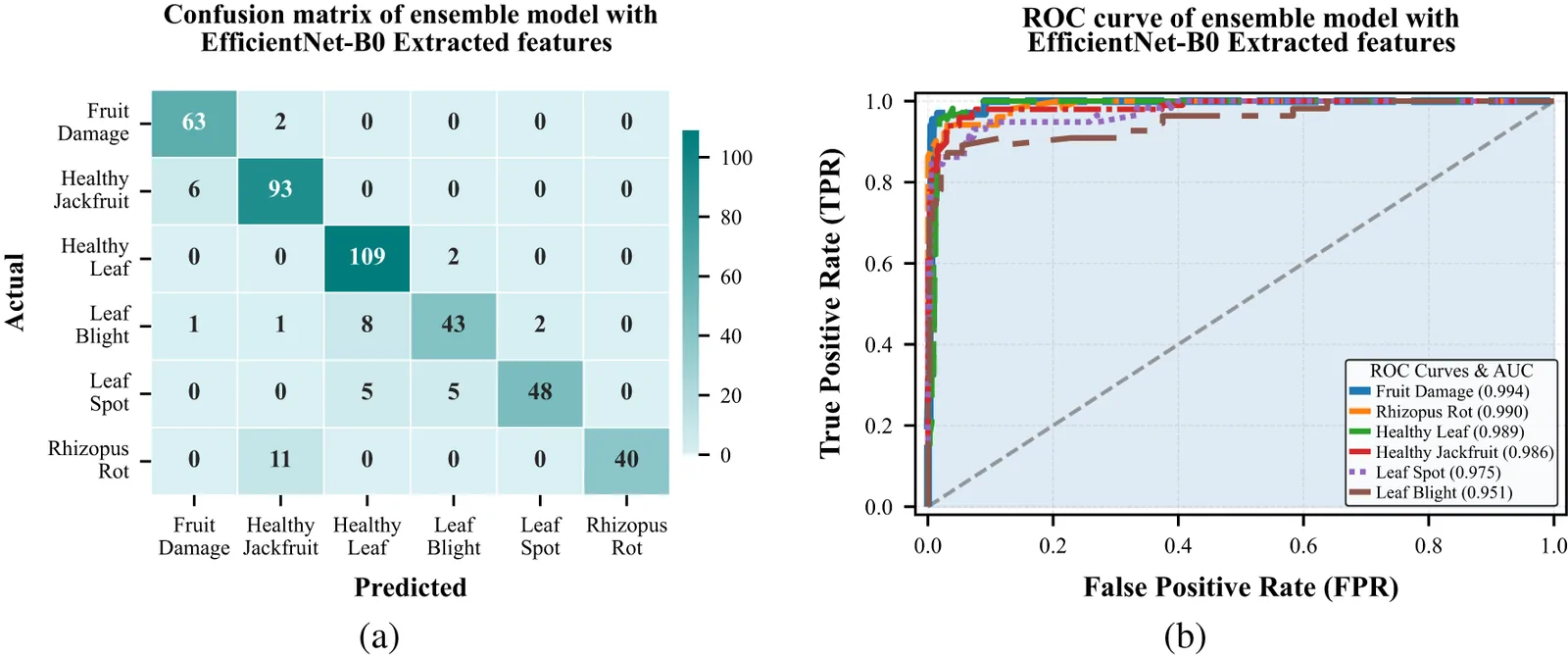
Performance visualization of the ensemble model with EfficientNet-B0-extracted features: **(a)** confusion matrix and **(b)** ROC curve.

#### 4.2.5 SMOTE-based balancing and PCA reduction of EfficientNet-B0 features for machine learning models

All machine learning models' classification performance significantly improved when SMOTE was used for class balancing and PCA was used to reduce dimensionality on the EfficientNet-B0 extracted features. With weighted F1 scores of 95.81% and balanced macro, RF achieved the highest single model accuracy of 95.81%. XGBoost achieved 94.76% accuracy with a great precision-recall balance (macro F1 score of 94.73%), where the SVM model came in second with 95.66% accuracy and a weighted F1-score of 95.65%. KNN achieved 86.83% accuracy with a macro F1-score of 86.48%, whereas CatBoost achieved 91.92% accuracy with a weighted F1-score of 91.87%. With a 96.71% accuracy rate and macro averaged precision, recall, and F1-score of 96.74%, 96.70%, and 96.70% respectively, the ensemble model significantly beat all individual classifiers. These findings demonstrate that combining EfficientNet-B0 features with SMOTE and PCA resulted in more balanced and robust representations, allowing for consistent performance improvements across a range of classifiers. The performance comparison is presented in Table 13 for enhanced comprehension.

**Table 13:** Performance of ML algorithms with SMOTE-based balancing and PCA-reduced EfficientNet-B0 features.

Model	Precision	Recall	F1-score	Accuracy
RF	95.94	95.80	95.81	95.81
SVM	95.71	95.65	95.64	95.66
XGBoost	94.84	94.75	94.73	94.76
CatBoost	92.15	91.90	91.86	91.92
**Ensemble model**	**96.74**	**96.70**	**96.70**	**96.71**

Bold values indicate the best-performing model.

The ROC curve and confusion matrix with SMOTE-balanced and PCA-reduced EfficientNet-B0 features are illustrated in Figure 9.

**Figure 9:**
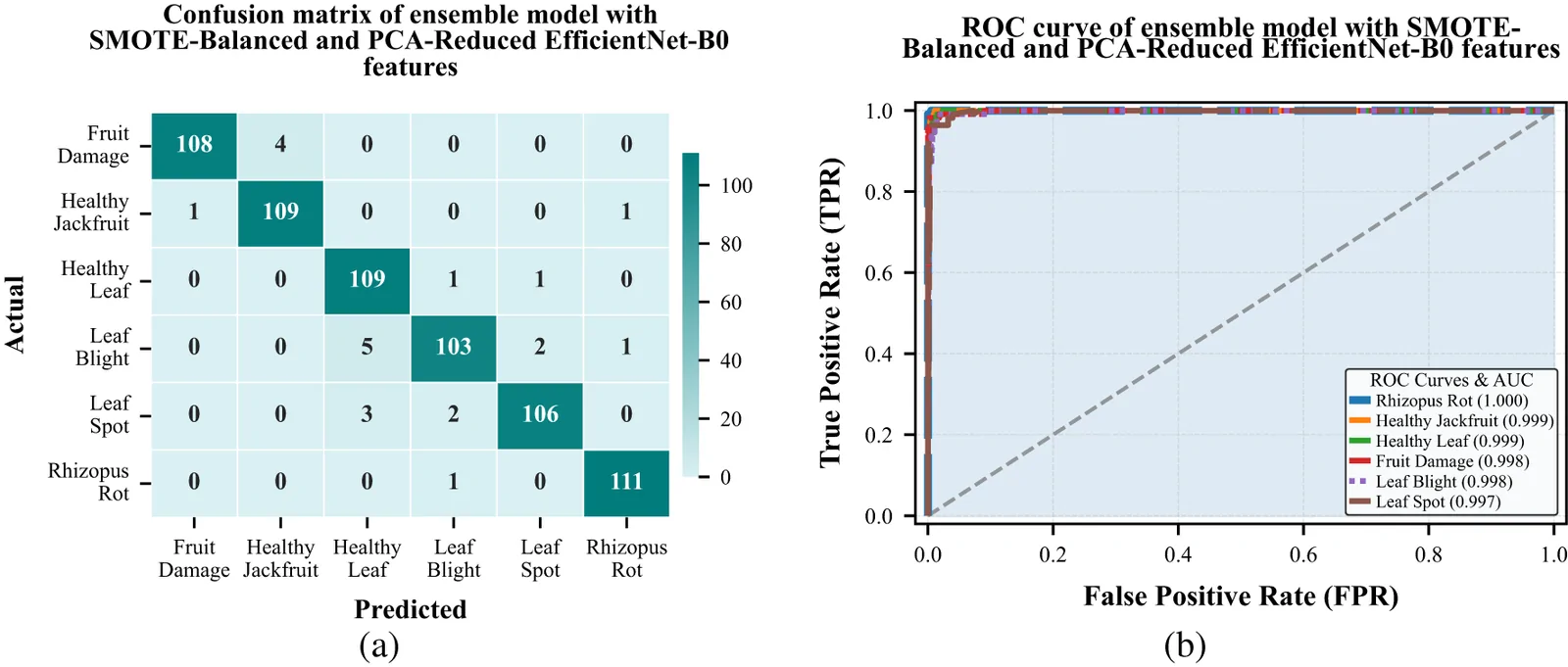
Performance visualization of the ensemble model with SMOTE-balanced and PCA-reduced EfficientNet-B0 features: **(a)** confusion matrix and **(b)** ROC curve.

#### 4.2.6 Machine learning algorithms in the augmented dataset

The models consistently produced strong results across all classes when direct machine learning was applied to the augmented dataset. With an F1-score of 92.0% and an accuracy of 90.4%, RF showed balanced performance across classes. With an F1-score of 95.3% and an accuracy of 94.2%, SVM achieved the best result among the individual classifiers. XGBoost recorded an F1-score of 92.9% and an accuracy of 91.6%, while CatBoost achieved a comparable F1-score of 91.0% and an accuracy of 90.4%. KNN recorded the lowest performance, with an F1-score of 86.5% and an accuracy of 88.0%. The ensemble model outperformed every individual classifier, achieving an F1-score of 95.9% and an accuracy of 95.4%. These results demonstrate that using PCA reduction in conjunction with direct machine learning on enhanced data yields strong classification results, with the ensemble technique showing the best consistent performance. The performance comparison is presented in Table 14 for better comprehension.

**Table 14:** Performance of machine learning algorithms on the augmented dataset.

Model	Precision	Recall	F1-score	Accuracy
RF	88.6	95.7	92.0	90.4
SVM	93.3	97.3	95.3	94.2
XGBoost	90.5	95.3	92.9	91.6
CatBoost	89.4	92.7	91.0	90.4
KNN	79.2	95.3	86.5	88.0
**Ensemble model**	**94.2**	**97.7**	**95.9**	**95.4**

Bold values indicate the best-performing model.

The ROC curve and confusion matrix of the ensemble model on the augmented dataset are presented in Figure 10.

**Figure 10:**
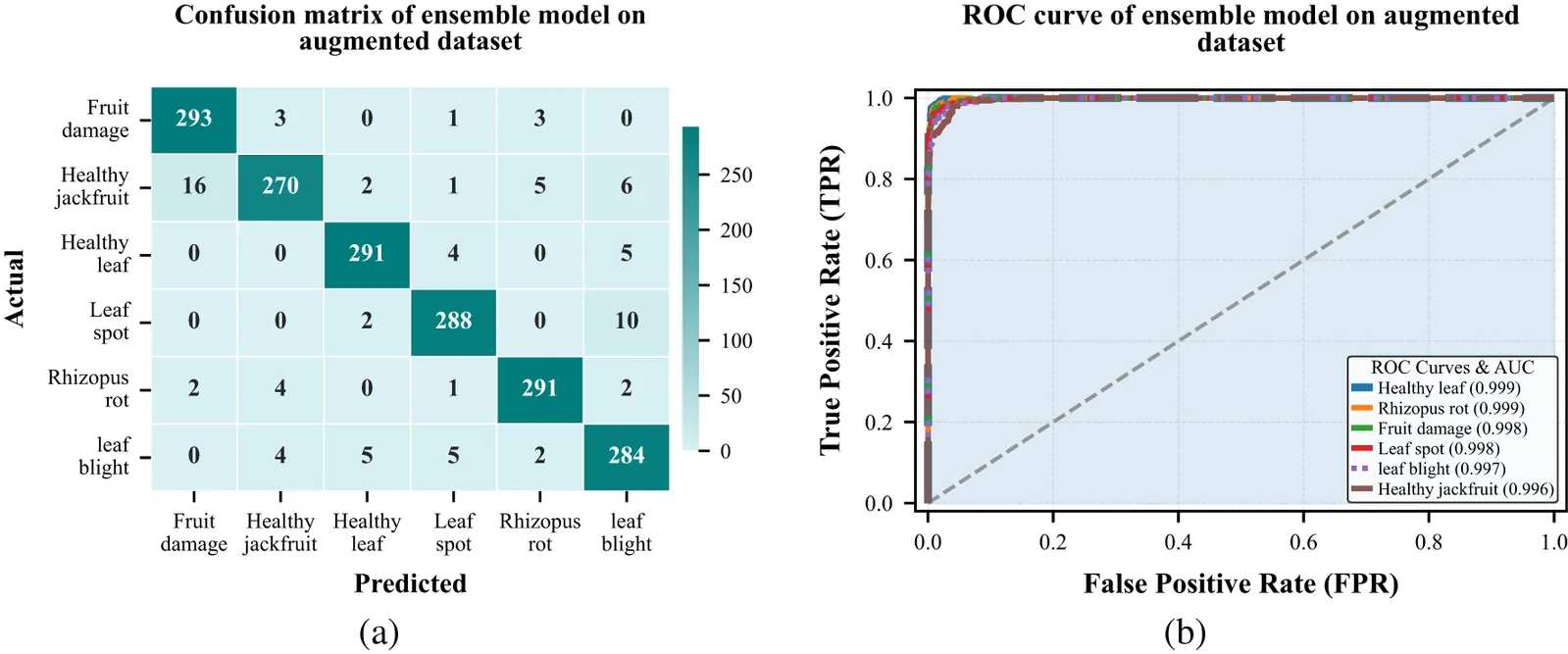
Performance visualization of the ensemble model on the augmented dataset: **(a)** confusion matrix and **(b)** ROC curve.

#### 4.2.7 Feature extraction using a custom CNN applied PCA with machine learning classification

All classifiers showed further improvement when feature extraction was performed using the custom CNN on the augmented dataset and reduced with PCA. RF achieved an F1-score of 98.40% and an accuracy of 98.41%. SVM followed with an F1-score of 98.75% and an accuracy of 98.74%, the strongest result among the individual classifiers. XGBoost achieved an F1-score of 98.29% and an accuracy of 98.30%, while CatBoost recorded an F1-score of 98.00% and an accuracy of 98.00%. KNN achieved the lowest individual result, with an F1-score of 97.80% and an accuracy of 97.80%. Every individual model was surpassed by the ensemble strategy, which achieved 99.39% accuracy with macro-averaged precision, recall, and F1-score of 99.28%, 99.27%, and 99.28%, respectively. Fruit damage (F1 = 99.83%), healthy jackfruit (F1 = 99.50%), healthy leaf (F1 = 99.00%), leaf spot (F1 = 99.67%), rhizopus rot (F1 = 99.67%) and leaf blight (F1 = 98.66%) were the ensemble's consistently high class-wise findings. These numerical enhancements unequivocally show that augmentation results in highly discriminative representations when paired with PCA and custom CNN based feature extraction, allowing for nearly flawless classification across all six classes. The performance comparison is presented in Table 15 for enhanced comprehension.

**Table 15:** Performance of machine learning algorithms with Custom CNN-extracted and PCA-reduced features.

Model	Precision	Recall	F1-score	Accuracy
RF	98.40	98.41	98.40	98.41
SVM	98.77	98.74	98.75	98.74
XGBoost	98.31	98.30	98.29	98.30
CatBoost	98.10	98.00	98.00	98.00
KNN	97.90	97.80	97.80	97.80
**Ensemble model**	**99.28**	**99.27**	**99.28**	**99.39**

Bold values indicate the best-performing model.

Figure 11 show the ROC curve and confusion matrix obtained using PCA-reduced custom CNN features on the augmented dataset.

**Figure 11:**
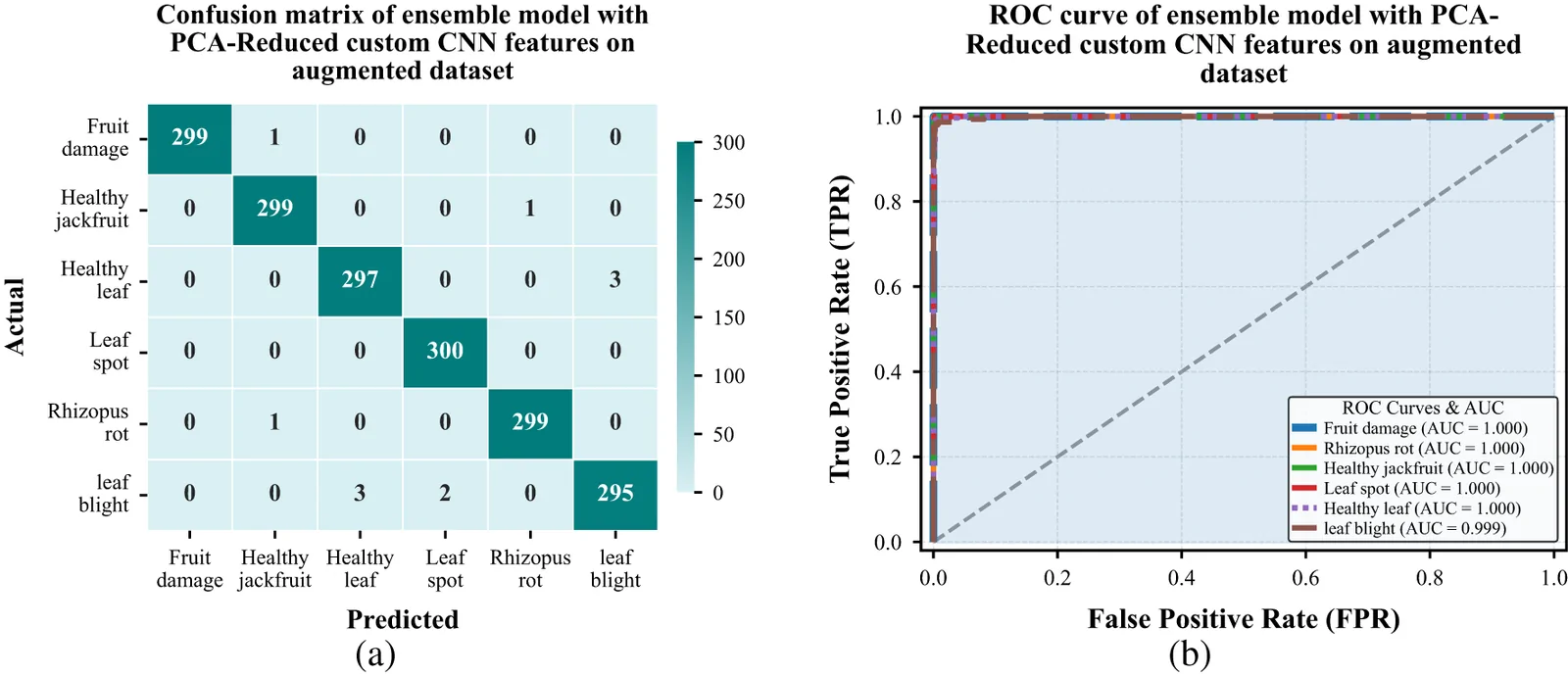
Performance visualization of the ensemble model with PCA-reduced custom CNN features on the augmented dataset: **(a)** confusion matrix and **(b)** ROC curve.

In conclusion, the outcome improved from 75.5 to 99.39%, reflecting a 33.64% enhancement in machine learning through the utilization of a combination of extracted features and machine learning models. This is far more economical than the deep learning model. The comparative histogram is presented in Figure 12 below for enhanced visual clarity.

**Figure 12:**
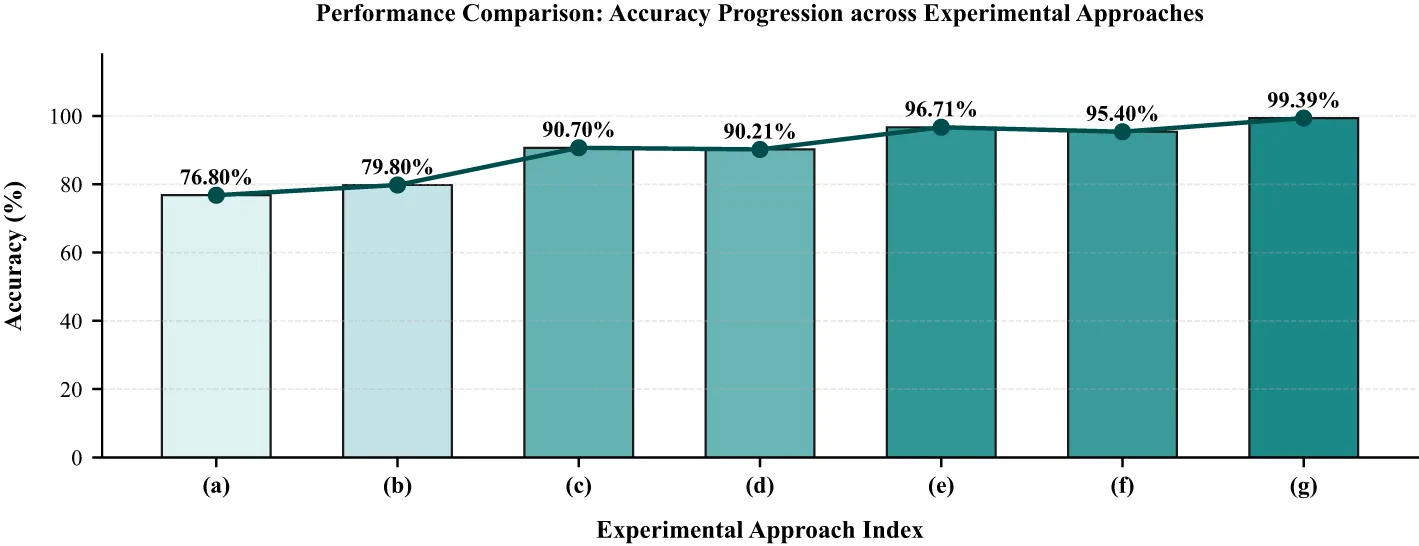
Histogram of accuracy progression across ensemble model approaches: **(a)** ML on raw dataset, **(b)** custom CNN feature extraction, **(c)** SMOTE + PCA on custom CNN features, **(d)** EfficientNet-B0 feature extraction, **(e)** SMOTE + PCA on EfficientNet-B0 features, **(f)** ML on augmented dataset, and **(g)** custom CNN + PCA on augmented dataset.

### 4.3 Ablation studies

To demonstrate the gradual advancements in architecture, calibration, and fusion method, four sample ensemble versions were chosen. By implementing controlled modification, each configuration improved on the one before it, increasing accuracy over time from 98.97 to 99.39%. RF, SVM, XGBoost, CatBoost, and KNN were the same base classifiers used in all models, and they were all trained on the same dataset split (6,300 training, 900 validation, and 1,800 test images).

#### 4.3.1 Ablation study V1: preliminary stacking

In the initial setup, a stacking ensemble was utilized, in which a basic meta-learning classifier (written with a typical ML algorithm like gradient boosting) combined the calibrated probability outputs from the four base models. None of the temperature scaling nor weight optimization were used. This baseline demonstrated limited calibration and modest overconfidence in some classes, especially Healthy Leaf and Leaf Blight, but it provided a strong foundation with 98.97% accuracy and 0.988 macro-F1.

#### 4.3.2 Ablation study V2: probability blending

Pure stacking was replaced with a weighted probability blending technique to increase model synergy. The discriminative reliability of each model was evaluated by optimizing its weights based on validation F1-scores, which were KNN 0.53, SVM 0.23, XGBoost 0.16, and CatBoost 0.08. Probability distributions were normalized using temperature scaling. With this adjustment, performance improved to 0.9927 macro-F1 and 99.00% accuracy, proving that blending calibrated outputs produces higher class-wise consistency than unweighted stacking.

#### 4.3.3 Ablation study V3: calibrated meta learner fusion

This version included systematic temperature calibration for each base classifier to the meta learner, which increased reliability and smoothed confidence outputs across classes. The process design remained the same; however, calibration parameters were adjusted based on validation loss. The revised setup improved the recall-precision balance and decreased bias in minority classes by achieving 99.22% accuracy and 0.9930 macro F1.

#### 4.3.4 Ablation study V4: final calibrated blended ensemble

The final structure included ensemble-level probability averaging, calibrated meta-learning, and optimal weighted blending. On the 1,800 image test set, this version performed the best, with 99.39% accuracy, 0.9939 macro-F1, and micro-AUC 1.00. There were very few incorrect classifications between healthy and leaf blight in the confusion matrix (Figure 9), which showed strong diagonal dominance. Near-perfect separability was validated by the Precision-Recall and ROC curves, with AUC of 1.00 for five classes and 0.999 for one.

The detailed configuration parameters of the four ensemble model versions are summarized in Table 16.

**Table 16:** Parameter grid of ensemble versions (V1–V4).

Version	Temperature values	Meta learner (best params)	Blend weights
V1	None	XGBoost(meta), n_estimators = 200, max_depth = 4, learning_rate = 0.05	None
V2	SVM = 0.8, XGB = 1.0, RF = 1.0, KNN = 0.5, Cat = 1.0	XGBoost(meta), n_estimators = 500, max_depth = 5, eta = 0.05	SVM = 0.4, XGB = 0.0, RF = 0.1, KNN = 0.2, Cat = 0.3
V3	SVM = 0.8, XGB = 1.0, RF = 1.0, KNN = 0.5, Cat = 1.0	MLPClassifier, hidden = (128, 64), activation = relu, max_iter = 500	RF = 1.0, others ≈ 0.0
V4	Learned per-model via NLL minimization	XGBoost(meta), n_estimators = 800, max_depth = 6, learning_rate = 0.05	Auto-optimized (∑*w* = 1)

The accuracy progression for each of the four ensemble versions is illustrated in Figure 13, which shows that improved calibration and fusing techniques consistently increase performance.

**Figure 13:**
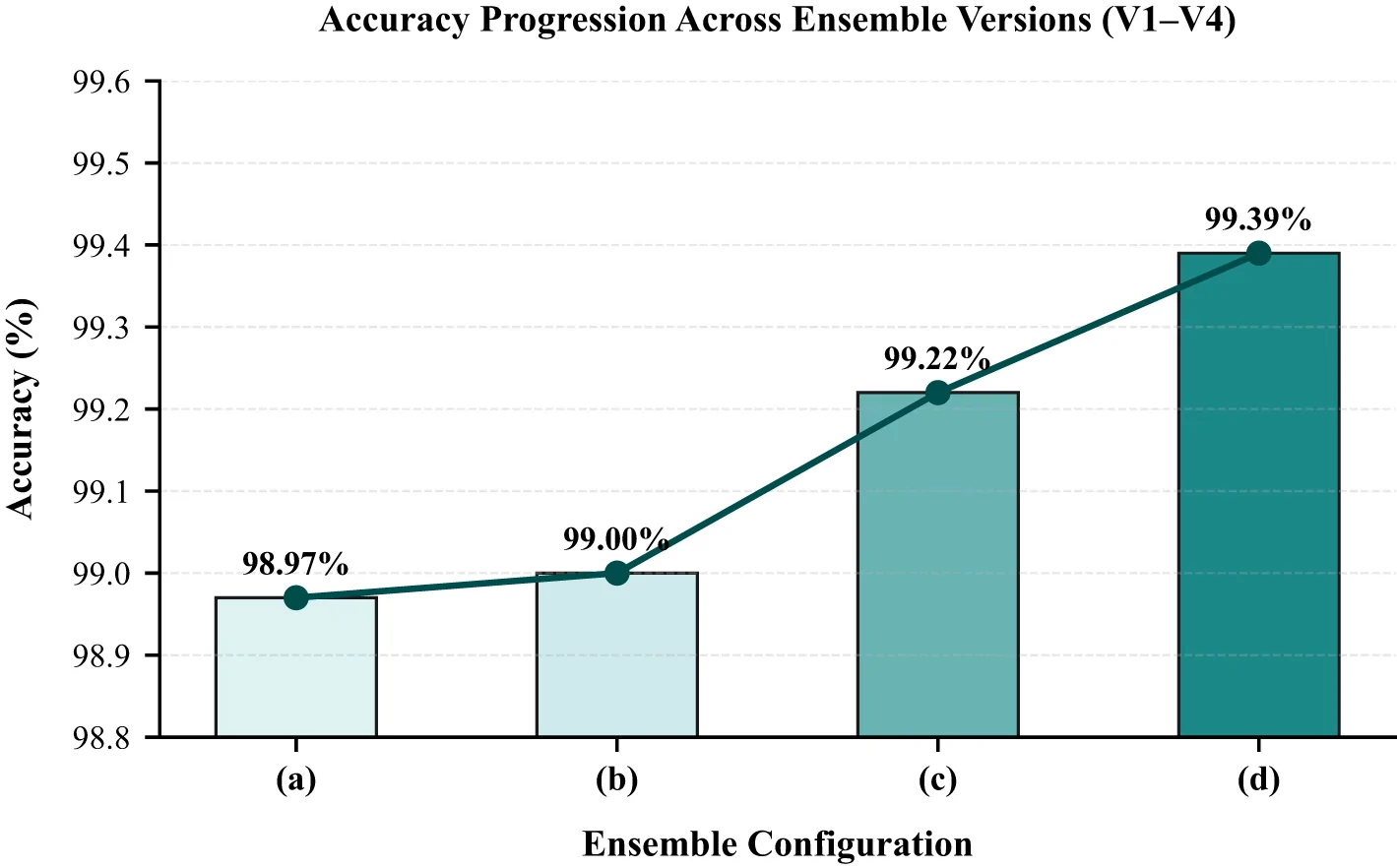
Accuracy progression across ensemble versions (V1–V4). The 0.42-point accuracy gain and 0.59-point macro-F1 gain from V1 to V4 isolate the contribution of calibration and weighted blending independent of any change in base classifiers or data.

The progressive enhancements in the four ensemble versions demonstrate the cumulative impact of meticulous calibration and weighted integration on prediction stability. The shift from uncalibrated stacking (V1) to adaptively calibrated blending (V4) resulted in an increase of +0.42% in accuracy and +0.59% in macro-F1. This machine learning-based architecture attained near-perfect performance through meticulous probability calibration, weight adjustment, and ensemble-level averaging, demonstrating that well-optimized classical methods can compete with or exceed more complex deep learning models for structured visual feature sets.

### 4.4 Comparative analysis

A summary of recent studies on plant disease classification shows that existing accuracy rates typically range from 95.00 to 98.38%, while the suggested hybrid CNN-ML architecture achieves 99.39%, demonstrating a significant performance improvement. A comparison with previous jackfruit studies is presented in Table 17. The jackfruit fruit damage classification model in Oraño et al. (2020) achieves an accuracy of about 93.42%. It lacks deep representation and only uses handcrafted texture features, but it provides domain relevance. Despite achieving 89.59% accuracy, the ML-based method in Habib et al. (2020) excludes deep learning-based features which limits its scalability across visual changes. Although it does not incorporate hybrid or ensemble refinement and instead classifies fruit species rather than diseases, a more comprehensive transfer learning process in Sumari et al. (2022) achieves 96.33% accuracy. The customized CNN technique is limited by the size of the dataset and lacks feature engineering yet it offers 95% accuracy while preserving task relevance across tree sections (Gomathi et al., 2023). In the meantime, the attention-enhanced deep model in Gunasekaran and Thambusamy (2025) achieves a strong 99.25% accuracy but it has a larger computational footprint that makes it inappropriate for deployment with limited resources. The proposed approach incorporates numerous significant enhancements such as feature compression, imbalance management, and calibrated fusion into an effective architecture. This standardized pipeline enhances prediction consistency across all categories while simultaneously decreasing processing demands, hence increasing its suitability for limited-resource or edge computing settings. The achieved accuracy of 99.39% signifies a significant improvement over current solutions, establishing an improved trade-off among accuracy, efficiency, and reliability compared to the examined methods.

**Table 17:** Comparative analysis of recent studies.

Author(s)	Method	Accuracy	Remarks
Sumari et al. (2022)	VGG16 (TL-based)	96.33%	Species level classification
Habib et al. (2020)	RF and SVM (ML)	89.59%	No deep features
Oraño et al. (2020)	BPNN with texture features	93.42%	Handcrafted features only
Gomathi et al. (2023)	Custom CNN	95.00%	Limited dataset
Gunasekaran and Thambusamy (2025)	Improved ResNet with SE blocks	99.25%	High compute cost
**This study**	**Proposed model**	**99.39%**	**Resource efficient**

Bold indicates the present study.

### 4.5 Theoretical complexity and resource-efficiency evaluation

To provide an analytical foundation for the claimed resource efficiency of the proposed framework and address contemporary edge-computing baselines, we formalize the parametric footprint of the proposed ensemble model's classification head. Let *M* represent the number of base estimators integrated within the primary stacking layer (*M* = 5), and let *C* denote the cardinal number of target crop conditions (*C* = 6).

Decoupling high-dimensional deep feature processing from our final classification task maps predictions into a compressed meta-feature matrix $X_{\text{meta}}\in {\mathbb{R}}^{N\times F}$. The dimensionality of this meta-space (*F*) incorporates the base class probability distributions combined with higher-order certainty metrics consisting of Shannon Entropy vectors (*M*_entropy_ = 5) and Margin Premiums (*M*_margin_ = 5), the latter grounded in the theoretical link between voting-margin distributions and ensemble generalization established by Schapire et al. (1998), as given in Equation 1:


$$\begin{array}{l} F=\left(M\times C\right)+M_{\text{entropy}}+M_{\text{margin}}=\left(5\times 6\right)+5+5=40 \end{array}$$ (1)

The downstream Multi-Layer Perceptron (MLP) meta-learner utilizes dual hidden layers configured at *H*_1_ = 128 and *H*_2_ = 64 hidden nodes. The total number of trainable weight and bias parameters (|Θ|) within this decision boundary is mathematically defined following the standard fully-connected layer parameterization convention (Sze et al., 2017), as defined in Equation 2:


$$\begin{array}{l} |\Theta |=\overset{2}{\underset{j=1}{\sum }}\left(H_{j-1}\cdot H_{j}+H_{j}\right)+\left(H_{2}\cdot C+C\right) \end{array}$$ (2)

where *H*_0_ = *F* = 40. Evaluating this layout explicitly yields Equation 3:


$$\begin{array}{l} |\Theta |=\left(40\times 128+128\right)+\left(128\times 64+64\right)+\left(64\times 6+6\right)\\ \;=\;5,248+8,256+390=13,894 \end{array}$$ (3)

This parameter footprint (13, 894 parameters) represents a major structural reduction compared to the linear classification layers of standard edge transformers when configured for large target environments [such as DeiT-Tiny's original classification head targeting standard ImageNet counts at ~193, 000 parameters, derived from its 192-dimensional embedding space (Touvron et al., 2021)] or heavy dense classification matrices, which typically scale up into the millions. Comparable lightweight hybrid architectures for leaf disease detection report similar accuracy–efficiency trade-offs at substantially higher parameter cost; for example, the Swift-Lite-CvT convolution–vision-transformer model achieves 93.51% accuracy on tomato leaf images with plain and complex backgrounds using a 5.8 MB footprint and 44-min training time—still orders of magnitude heavier than the 13,894-parameter decision head proposed here (Gangwar et al., 2024). This structural efficiency explains our framework's sub-millisecond average inference latency range (τ = 0.4196 − 0.8922 ms per sample).

Furthermore, while modern transformers face quadratic computational constraints ($O\left(N^{2}\right)$) due to their multi-head self-attention mechanisms (Tay et al., 2022), our hybrid framework operates within linear spatial boundaries (O(N)). This mathematically confirms its suitability for low-power microcontrollers and practical smart farming deployment (Ray, 2022).

### 4.6 Cross-dataset validation and domain generalization bounds

To evaluate the generalization capability of the proposed model and confirm its resilience against regional dataset bias, external validation was performed across two entirely independent target benchmarks:

External dataset 1: evaluated over a large-scale 3-class distribution (5, 124 samples) representing distinct geographical capture zones from the public dataset repository (Basak, 2023), the model achieved an outstanding 99.73% accuracy with a sub-millisecond average inference latency of 0.5043 ms per sample while managing only 11, 779 weights within the stacking layer. The corresponding empirical performance profiles, including the raw multi-class confusion matrix and the receiver operating characteristic (ROC) curves, are illustrated in Figures 14a, b, respectively.

**Figure 14:**
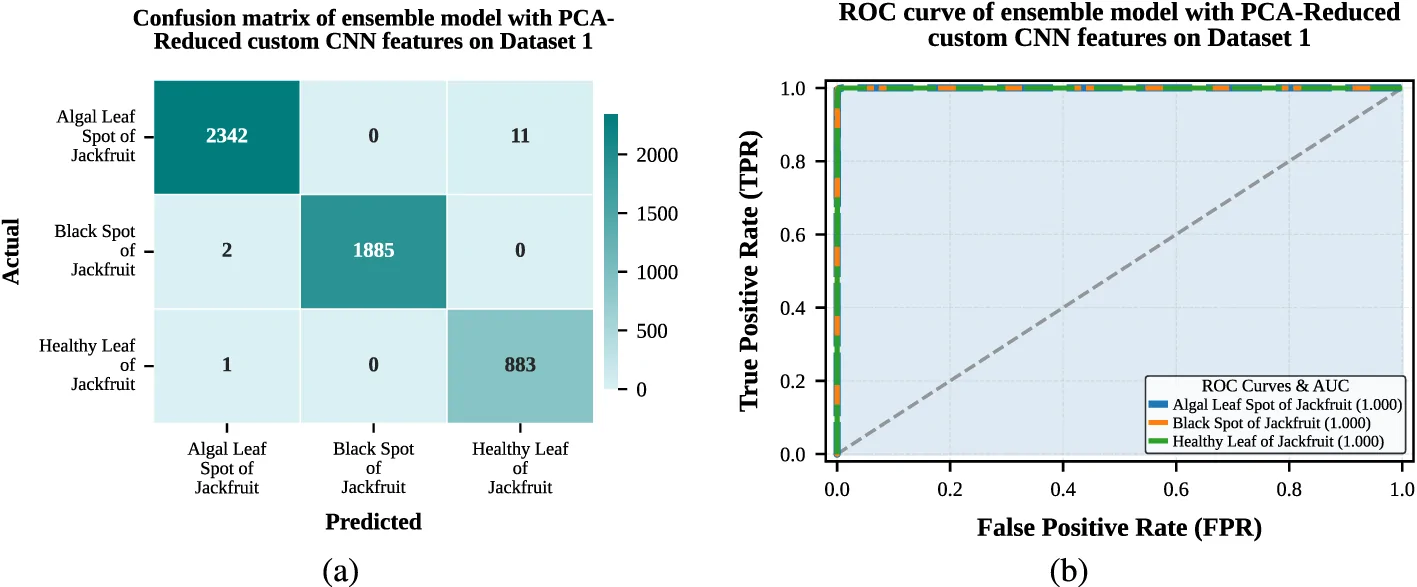
Multi-class diagnostic evaluation profiles of the ensemble model over the pristine, out-of-sample test partition of external dataset 1: **(a)** raw integer count confusion matrix and **(b)** multi-class receiver operating characteristic (ROC) curves.

External dataset 2: evaluated over a challenging eight-class profile (1, 437 test samples) captured using low-cost mobile sensors under varying field conditions sourced from the peer-reviewed Mendeley public repository (Hossain et al., 2025), the framework maintained a highly stable 92.41% accuracy with an average latency of 0.3785 ms per sample backed by a highly optimized 15, 304 parameter decision head. The corresponding empirical performance profiles, including the multi-class raw confusion matrix and the receiver operating characteristic (ROC) curves, are illustrated in Figures 15a, b, respectively.

**Figure 15:**
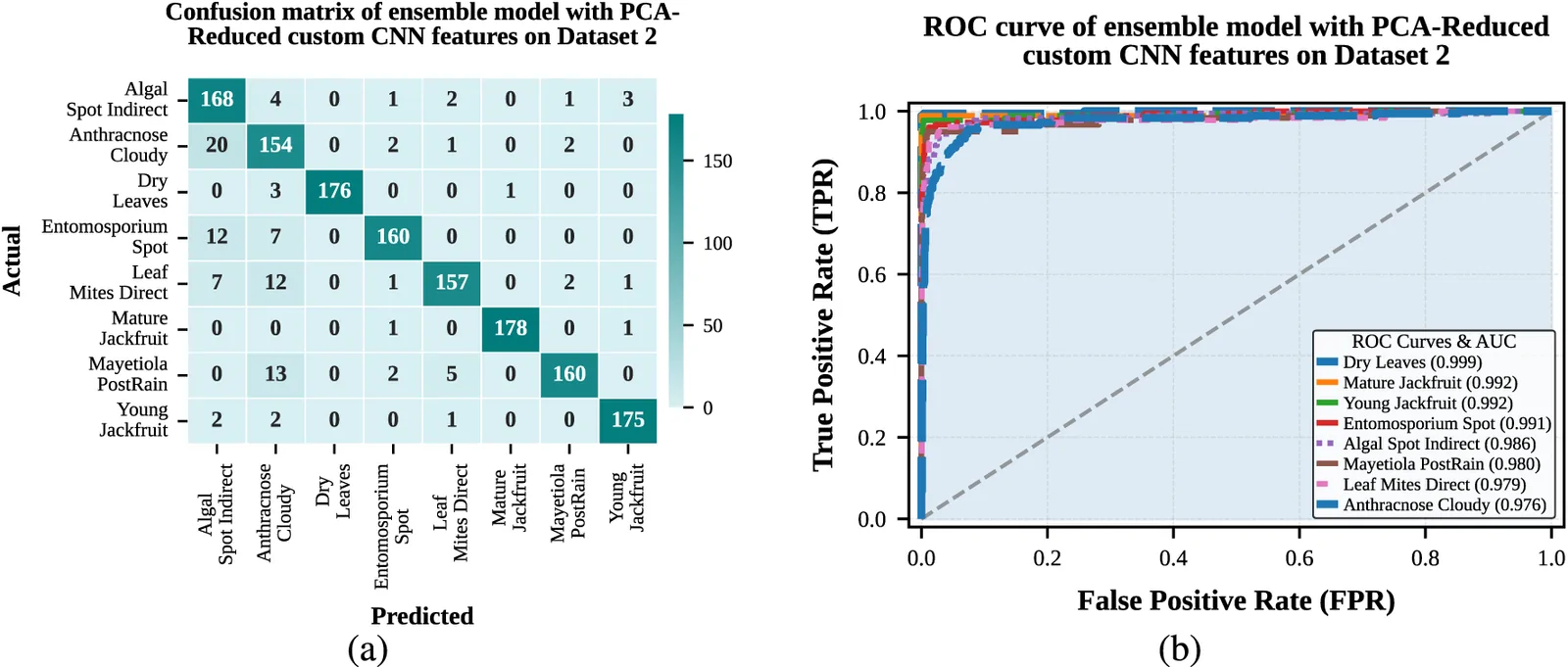
Multi-class diagnostic evaluation profiles of the ensemble model over the pristine, out-of-sample test partition of external dataset 2: **(a)** raw integer count confusion matrix and **(b)** multi-class receiver operating characteristic (ROC) curves.

The model's ability to maintain high precision and recall boundaries across these diverse datasets demonstrates that it generalizes effectively beyond its original training distribution. By extracting robust visual features and projecting them into a stable, low-dimensional meta-feature space, the architecture proves resilient against hardware variations, environmental lighting changes and background crop clutter. This performance beyond the sample confirms the framework's readiness for real-world farming infrastructure and embedded agricultural monitoring systems.

### 4.7 Statistical robustness and confidence bounds

To quantify the precision of the reported accuracies without introducing new experimental runs, we computed 95% Wilson confidence intervals (Wilson, 1927) directly from the sample sizes and correct-classification counts underlying the confusion matrices already reported in Table 15, Figures 14, 15, summarized in Table 18. This approach follows recent practice in the plant disease classification literature, where confidence intervals have similarly been used to confirm that high (99%+) classification scores are statistically grounded rather than artifacts of overfitting or data leakage (Murugesan et al., 2025). The headline result is tightly bounded [99.39%, 95% CI (98.91%, 99.66%)], and both external benchmarks show similarly narrow intervals, indicating the reported accuracies are not artifacts of a small or favorably sampled test set.

**Table 18:** 95% Wilson confidence intervals on reported test accuracy, computed directly from the published confusion matrices.

Evaluation	Correct/total	Accuracy	95% CI
Main test set	1,789/1,800	99.39%	[98.91%, 99.66%]
External dataset 1	5,110/5,124	99.73%	[99.54%, 99.84%]
External dataset 2	1,328/1,437	92.41%	[90.93%, 93.67%]

Beyond these bounds, robustness is further supported by the narrow 0.42-point accuracy spread observed across four independently calibrated ensemble configurations in the ablation study (Section 4.3) and by consistent performance across the two independent external benchmarks (Section 4.6), together indicating low variance in the reported result.

To further characterize where the model's residual uncertainty lies, we additionally computed class-wise recall with 95% Wilson confidence intervals directly from the diagonal and row totals of the confusion matrix in Figure 11, summarized in Table 19. All six classes show recall above 98% with intervals that remain narrow despite the smaller per-class sample size (*n* = 300), with Leaf Blight showing the widest interval, consistent with its slightly lower diagonal count in the confusion matrix.

**Table 19:** Class-wise recall with 95% Wilson confidence intervals for the ensemble model on the main test set (*n* = 300 per class), computed directly from the confusion matrix in Figure 11.

Class	Correct/total	Recall	95% CI
Fruit damage	299/300	99.67%	[98.14%, 99.94%]
Healthy jackfruit	299/300	99.67%	[98.14%, 99.94%]
Healthy leaf	297/300	99.00%	[97.10%, 99.66%]
Leaf spot	300/300	100.00%	[98.74%, 100.00%]
Rhizopus rot	299/300	99.67%	[98.14%, 99.94%]
Leaf blight	295/300	98.33%	[96.16%, 99.29%]

## 5 Discussion

RQ1 discusses the performance of traditional machine learning models on raw jackfruit images without any form of feature extraction, which was the first stage of primary motivation. In line with RO1, we applied classifiers such as Random Forest, Support Vector Machine, XGBoost, CatBoost, and KNN directly to the raw dataset. The results showed that while these methods were computationally lightweight and fast, their accuracies were limited to around seventy percent, with some classes such as rhizopus rot and leaf spot being consistently misclassified. This confirmed that although traditional machine learning can provide a starting point, the lack of feature abstraction limits its capacity to discriminate complex disease patterns in jackfruit. Thus, RQ1 was answered with the understanding that raw machine learning pipelines are insufficient for reliable agricultural disease detection.

Building upon this baseline, we turned to RQ2, which investigated whether convolutional neural network-based feature extraction could improve classifier accuracy compared to direct machine learning models. We implemented a custom CNN and EfficientNetB0 to generate deep embeddings, which were then used by the same machine learning classifiers, guided by RO2. This transition markedly improved the results, with ensemble models reaching nearly eighty percent accuracy. The improvement validated that CNN could drop raw images into semantically meaningful features using shallow machine learning classifiers to perform far better than on raw images. However, it was also clear that imbalance in class and feature repetition continued to decrease performance, leaving room for further refinement.

This naturally led to RQ3, which asked how Principal Component Analysis (PCA) and Synthetic Minority Oversampling Technique (SMOTE) could help address the problems of high dimensionality and class imbalance. We used SMOTE to create synthetic samples for minority classes and PCA to compress feature vectors, as guided by RO3. These two methods functioned together to have a revolutionary effect. Reduced feature dimensionality allowed models to train more quickly, and in minority disease categories, they also achieved significantly higher recall and F1 scores. The fact that the ensemble accuracy above 90% showed that PCA and SMOTE combined offered a feasible and efficient way to address the problems caused by imbalanced agricultural datasets. RQ3 was consequently addressed with strong evidence that, when combined with contemporary deep embeddings, such classical techniques continue to be beneficial.

After addressing the issues of dimensionality and balance, in this research we turned our attention to RQ4. The impact of data augmentation on robustness and classification accuracy in field settings was the subject of this study. Following RO4, we applied a variety of augmentation techniques, including rotations, flips, skew transformations, and brightness adjustments, to simulate the diverse conditions under which jackfruit images are captured. The enriched dataset allowed the models to generalize better to unseen data and reduced the risk of overfitting. The results were improving when CNN features, PCA, SMOTE, and augmentation were combined, the ensemble model achieved accuracies exceeding 99.00%, with F1-scores near perfection across all six classes. The result offered a strong response to RQ4 by showing how the concept of augmentation not only helps but is a necessity in making AI in agriculture robust.

Our research outcome led us to the final question, RQ5, which examined whether the hybrid DL machine learning pipelines have a better cost-accuracy trade-off than independent machine learning or deep learning approaches. As part of RO5, we used EfficientNet-B0 as an extractor of features and combined it with classical machine learning classifiers augmented with PCA and SMOTE. The hybrid model attained 96.71% accuracy and balanced macro F1 scores of 96.00%, which were very high when compared to both single machine learning and end-to-end deep learning. Notably, the calculation cost was less as compared to training complete deep learning networks, so the framework is much more feasible in resource-limited settings. This gave a final response to RQ5 and proved that not only are hybrid pipelines competitive but constitute the most economical solution to crop disease classification.

The development between the RQ1 and RQ5 in total describes a logical process of incremental advancement. With out-of-the-box machine learning on raw data, we exposed its ineptitude and shifted to CNN-based feature extraction which refined the representation but did not address the problem of imbalance. The combination of PCA and SMOTE allowed us to minimize redundancy and offer balanced learning as well as further augmentation to withstand real-world variability. Lastly, hybrid deep learning and machine learning pipelines and ensemble learning provided the most desirable balance between accuracy and computational efficiency. Each stage addressed a specific research question and fulfilled the associated objective, resulting in an almost perfect classification system that remains practical for smallholder agricultural settings.

Looking to the future, several directions arise from this study. First, enlarging the dataset across multiple places, varieties, and seasons will be necessary to validate model generalization under large-scale scenarios. Second, web deployment should be prioritized, using lightweight architectures, quantization, and pruning to ensure real-time disease detection on farmers' smartphones. Third, AI techniques such as Explainable AI techniques such as gradient-weighted class activation mapping (Grad-CAM) or SHapley Additive exPlanations (SHAP) could provide detailed overviews that can gain trust by allowing farmers and agronomists to see why a prediction was made. Fourth, integration with IoT-based smart farming systems could create comprehensive early warning platforms that combine image recognition with sensors, consistent with recent reviews highlighting the convergence of IoT and deep learning architectures in plant disease detection and classification (Dhaka et al., 2023). Fifth, the validated hybrid approach should be extended to other major crops such as mango, banana, tomato, and coffee, where AI-driven pipelines combining disease diagnosis with downstream quality assessment are an active and growing research area, as demonstrated in recent comprehensive systematic reviews (Solis Pino and Caicedo Apraez, 2025). Finally, emerging models such as self-supervised and few-shot learning should be evaluated to further reduce dependence on large, annotated datasets, making the system more adaptable to new diseases and crops.

## 6 Conclusion

This study addresses major challenges of AI in agriculture with a special focus on developing an accessible solution to diagnosing jackfruit diseases and quality evaluation, particularly in terms of strained farms. The study progressed from conventional machine learning methods to a hybrid framework integrating deep learning and machine learning, attaining a 99.39% accuracy in classification. This approach is a synergy between the strength of deep learning and the performance of machine learning models, making it affordable for smallholder farmers. The results show the possibility of inclusive AI approaches to enhance food security and reduce the livelihoods of farmers. Further developments will encompass a wider range of datasets, the optimization of models for portable devices, and the integration of image analysis with IoT management. The study attempts to encourage the broad adoption of technology and ensure that the advantages of AI are readily accessible to agricultural practitioners.

## Data Availability

The original jackfruit dataset collected for this study is available at https://data.mendeley.com/datasets/pt647jfn52/1. The external datasets analyzed in this study are publicly available at https://www.kaggle.com/datasets/shuvokumarbasak4004/jackfruit-leaf-diseases (Basak, 2023) and in the Mendeley Data repository (Hossain et al., 2025).
